# Cellular senescence in cancer: from mechanisms to detection

**DOI:** 10.1002/1878-0261.12807

**Published:** 2020-10-22

**Authors:** Hui‐Ling Ou, Reuben Hoffmann, Cristina González‐López, Gary J. Doherty, James E. Korkola, Daniel Muñoz‐Espín

**Affiliations:** ^1^ CRUK Cambridge Centre Early Detection Programme Department of Oncology Hutchison/MRC Research Centre University of Cambridge UK; ^2^ Department of Biomedical Engineering Knight Cancer Institute OHSU Center for Spatial Systems Biomedicine Oregon Health and Science University Portland OR USA; ^3^ Department of Oncology Cambridge University Hospitals NHS Foundation Trust Cambridge Biomedical Campus UK

**Keywords:** cancer, cellular senescence, detection, senoprobes, tumour microenvironment

## Abstract

Senescence refers to a cellular state featuring a stable cell‐cycle arrest triggered in response to stress. This response also involves other distinct morphological and intracellular changes including alterations in gene expression and epigenetic modifications, elevated macromolecular damage, metabolism deregulation and a complex pro‐inflammatory secretory phenotype. The initial demonstration of oncogene‐induced senescence *in vitro* established senescence as an important tumour‐suppressive mechanism, in addition to apoptosis. Senescence not only halts the proliferation of premalignant cells but also facilitates the clearance of affected cells through immunosurveillance. Failure to clear senescent cells owing to deficient immunosurveillance may, however, lead to a state of chronic inflammation that nurtures a pro‐tumorigenic microenvironment favouring cancer initiation, migration and metastasis. In addition, senescence is a response to post‐therapy genotoxic stress. Therefore, tracking the emergence of senescent cells becomes pivotal to detect potential pro‐tumorigenic events. Current protocols for the *in vivo* detection of senescence require the analysis of fixed or deep‐frozen tissues, despite a significant clinical need for real‐time bioimaging methods. Accuracy and efficiency of senescence detection are further hampered by a lack of universal and more specific senescence biomarkers. Recently, in an attempt to overcome these hurdles, an assortment of detection tools has been developed. These strategies all have significant potential for clinical utilisation and include flow cytometry combined with histo‐ or cytochemical approaches, nanoparticle‐based targeted delivery of imaging contrast agents, OFF‐ON fluorescent senoprobes, positron emission tomography senoprobes and analysis of circulating SASP factors, extracellular vesicles and cell‐free nucleic acids isolated from plasma. Here, we highlight the occurrence of senescence in neoplasia and advanced tumours, assess the impact of senescence on tumorigenesis and discuss how the ongoing development of senescence detection tools might improve early detection of multiple cancers and response to therapy in the near future.

## Graphical abstract

Cellular senescence is considered a crucial process for tumour suppression, which can be facilitated by immune surveillance. However, when senescent cells persist in tissues, they can also trigger a plethora of tumour‐promoting effects. Here, we discuss the main hallmarks, mechanisms and roles of senescence in cancer and provide a comprehensive revision of the available tools for its detection.

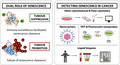

**Abbreviations**
5‐FU: 5‐fluorouracil
AAH: atypical adenomatous hyperplasia; AIS adenocarcinoma *in situ*
ATM: ataxia‐telangiectasia mutated
ATR: ATM‐ and Rad3‐related
B2M: β2‐microglobulin
BAX: BCL2‐associated protein X
BCL‐2: B‐cell lymphoma 2
BrdU: 5‐bromo‐2′‐deoxyuridine
C/EBPβ: CCAAT/enhancer‐binding protein beta
CCF: cytoplasmic chromatin fragment
CDK: cyclin‐dependent kinase
cfDNA: cell‐free DNA
cGAS‐STING: cyclic GMP‐AMP synthase linked to stimulator of interferon genes
CHK: checkpoint kinase
CIS: carcinoma innonbreakingspacesitu
CKI: CDK inhibitors
CM: conditioned medium
CSLC: cancer stem‐like cell
CT: computed tomography
CXCL: C‐X‐C‐motif ligand
DDR: DNA damage response
DSB: double‐strand breaks
EdU: 5‐ethynyl‐2′‐deoxyuridine
EMT: epithelial–mesenchymal transition
ER: endoplasmic reticulum
EV: extracellular vesicle
FFPE: formalin‐fixed and paraffin‐embedded
FOXO: forkhead box O
H3K20me3: trimethylation of lysine 20 on histone 3
H3K27ac: acetylation of lysine 27 on histone 3
H3K9me3: trimethylation of lysine 9 on histone 3
HER: human epidermal growth factor receptor
HMGB1: high mobility group box‐1
IL: interleukin
LMNB1: lamin B1
MAPK: mitogen‐activated protein kinase
MDM2: mouse double minute 2
MEK: MAPK/ERK kinase
MHC: major histocompatibility complex
MIA: minimally invasive adenocarcinoma
MMP: matrix metalloproteinase
MRI: magnetic resonance imaging
mTOR: mammalian target of rapamycin
nanoMIP: molecularly imprinted nanoparticle
NB: Nile blue
NF‐κB: nuclear factor kappa light‐chain enhancer of activated B cells
NIR: near‐infrared
NK: natural killer
NP: nanoparticle
NSCLC: non‐smallcell lung cancer
OIS: oncogene‐induced senescence
PDH: pyruvate dehydrogenase
PDK1: PDH‐inhibitory enzyme pyruvate dehydrogenase kinase 1
PDP2: PDH‐activating enzyme pyruvate dehydrogenase phosphatase 2
PDTX: patient‐derived tumour xenograft
PET: positron emission tomography
PI3K: phosphatidylinositol 3‐kinase
PTBP1: polypyrimidine tract binding protein 1
PTEN: phosphatase and tensin homolog
RB: retinoblastoma protein
ROS: reactive oxygen species
RPS14: ribosomal protein S14
SAHF: senescence‐associated heterochromatin foci
SASP: senescence‐associated secretory phenotype
SAβG: senescence‐associated β‐galactosidase
SBB: Sudan Black B
TGFβ: transforming growth factor β
TIF: telomere dysfunction‐induced foci
TIS: therapy‐induced senescence
TKI: tyrosine kinase inhibitor
TS: tumour suppressor
UPS: unfolded protein response
VEGF: vascular endothelial growth factor
γH2AX: phosphorylation of the histone H2AX

## 1 Cellular senescence: Introduction

The term senescence derives etymologically from *senex*, the Latin word for old. Based on the *in vitro* observation of the finite proliferation capacity of human fibroblasts upon serial cultivation, at the beginning of the 1960s Leonard Hayflick and Paul Moorhead introduced the concept of cellular senescence [1]. Such limited proliferative capacity was attributed to the gradual attrition, through multiple cell divisions, of the telomeres located at both ends of the chromosome, consisting of the repetitive TTAGGG DNA sequence as protective structures [2, 3]. This is referred to as replicative senescence [4]. Cellular senescence is a stable state of cell‐cycle arrest that is triggered in proliferative cells by multiple types of damage, including replicative stress, and is characterised by the implementation of a complex pro‐inflammatory secretory phenotype associated with altered metabolism (the so‐called ‘senescence‐associated secretory phenotype’, or SASP).

Cellular senescence is a very heterogeneous programme that varies depending on the different stimuli and cellular contexts to which it responds. Senescence is involved in several physiological and pathological processes, ageing and cancer being probably the most notorious. Cellular senescence has been characterised for over a half‐century, and a plethora of studies have proposed several types of senescence based on diverse stimuli [5]. While DNA damage‐induced senescence refers to the response to irreparable DNA damage caused by either endogenous sources [e.g., telomere shortening or double‐strand breaks (DSBs) occurring during proliferation], or exogenous sources (such as ionising radiation and DNA‐damaging agents), oncogene‐induced senescence (OIS) springs to action upon either the activation of oncogenes or the inactivation of tumour suppressors [6, 7]. In the context of cancer cells, therapy‐induced senescence (TIS) can emerge in response to a therapeutic regimen (e.g., cytotoxic chemotherapy or radiation) [8]. Furthermore, mitochondrial dysfunctionality and oxidative stress can also induce cellular senescence [9, 10]. Despite the fact that a diversity of stimuli can trigger it, cellular senescence exhibits certain distinct hallmarks that are not observed in other cellular states, providing promise for universal methods of detection and therapeutic targeting.

### 1.1 Hallmarks of senescence

**Fig. 1:**
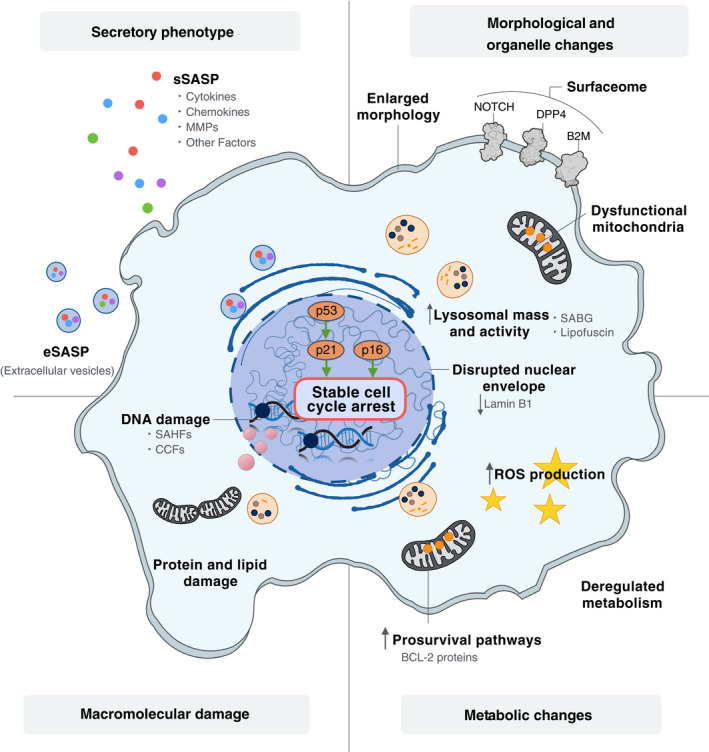
Hallmarks of cellular senescence. Senescence is triggered in response to a variety of stimuli, with senescent cells acquiring phenotypes derived from changes in morphology, the nucleus and the cytoplasm. B2M, β_2_ microglobulin; BCL‐2, B‐cell lymphoma 2; CCF, cytoplasmic chromatin fragment; DPP4, dipeptidyl‐peptidase 4; MMPs, matrix metalloproteinases; ROS, reactive oxygen species; SAβG, senescence‐associated β‐galactosidase; SAHF, senescence‐associated heterochromatin foci; SASP, senescence‐associated secretory phenotype.

Senescent cells usually acquire some structural changes as a result of stress‐induced signalling cascades, including flattened, enlarged and aberrant morphologies with modified cytoplasmic compositions. However, some characteristics of senescent cells (e.g., stable withdrawal from the cell‐cycle and specific morphological changes) are shared with other cellular states such as quiescence and terminal differentiation. The International Cell Senescence Association has proposed a consensus set of the defining hallmarks of senescence phenotypes based on the following four features: (a) cell‐cycle withdrawal; (b) macromolecular damage; (c) secretory phenotype, and (d) deregulated metabolism [11] (Fig. 1). Of note, while these are distinct cellular features, they have complex interplays and interdependence.

#### 1.1.1 Cell‐cycle withdrawal

Mammalian cell‐cycle progression is driven by the dynamics of cyclins and cyclin‐dependent kinases (CDKs), which ensure timely phase transition. Cyclin D/CDK4‐6 complexes promote cell‐cycle progression into G1, followed by cyclin E/CDK2 complexes eliciting G1/S phase transition; the progression into S phase and subsequent transition to S/G2 phase require competent cyclin A/CDK2 complexes; and cyclin B/CDK1 facilitates G2/M phase transition [12, 13]. Careful modulation of the activity of cyclin/CDKs by various CDK inhibitors (CKIs), as occurs in cellular quiescence and senescence, ensures efficient context‐dependent control of the cell cycle [14]. Increased levels of p27^KIP1^ mediate cell‐cycle arrest in G0 in quiescent cells [15, 16] while a decrease in p27^KIP1^ levels upon mitogenic stimulation reverses this arrest and leads to cell‐cycle re‐entry [17]. By contrast, the upregulation of p21^WAF1/CIP1^ (*CDKN1A*) and p16^INK4A^ (*CDKN2A*) commonly halts cell proliferation leading to cellular senescence. Accumulation of p21^WAF1/CIP1^ and p16^INK4A^ first leads to the hypo‐phosphorylation of retinoblastoma protein (RB) and then inhibits the transactivation of the E2F genes involved in nucleotide metabolism and DNA synthesis [18, 19], resulting in a stable cell‐cycle arrest [20].

Alongside the upregulation of CKIs, cellular senescence is also associated with some fundamental epigenetic changes [21, 22]. Distinct histone modifications, including elevated trimethylation of lysine 9 or lysine 20 on histone H3 (H3K9me3 or H3K20me3) may facilitate cell‐cycle arrest [21, 23], while acetylation of lysine 27 on histone H3 (H3K27ac) has a role in promoting the SASP [24]. Senescence‐associated heterochromatin foci (SAHF), which form to various extents in senescent cells depending on the stimuli used [23], are a gene repressing mechanism exerted through the induction of focal hypermethylation of DNA via cooperation between heterochromatin protein 1 (HP1) and DNA methyltransferase [25, 26]. The location of SAHF in the vicinity of E2F target genes implicated in cell‐cycle progression thereby imposes stable cell‐cycle withdrawal [23, 27, 28]. Additionally, loss of the nuclear structural protein lamin B1 (LMNB1) compromises the integrity of the nuclear envelope, leading to the production of cytoplasmic chromatin fragments (CCFs), which are involved in the regulation of the secretory phenotype of senescent cells [29]. Loss of LMNB1 and resultant CCFs are viewed as hallmarks of senescence [30, 31].

#### 1.1.2 Macromolecular damage

The substantial accumulation of macromolecular damage, such as DNA and protein damage, is another discriminating feature that distinguishes cellular senescence from cell differentiation. The progressive telomere shortening owing to successive cell divisions is eventually recognised and processed as DNA damage by the DNA damage response (DDR) machinery. When left unresolved, this induces cellular senescence [32]. In the case of OIS, oncogene‐driven hyperproliferation leads to the collision and subsequent collapse of replication forks, ultimately generating DSBs [33, 34], leading to induction of senescence. Senescent cells are metabolically active, and the accumulation of reactive oxygen species (ROS) contribute to oxidative DNA damage at telomeric G‐rich repeats, forming so‐called telomere dysfunction‐induced foci (TIFs) that are part of the DDR response [35, 37]. DDR processes lead to the phosphorylation of histones H2AX (γH2AX) and H3K9me3 which facilitate the binding and assembly of DNA repair machineries or proteins involved in the DDR signalling cascade. The detection of these modifications is widely used to identify DNA damage‐induced senescence [38, 39].

Furthermore, ROS oxidation of cysteine residues in protein tyrosine phosphatases and their subsequent removal by the proteasome‐dependent protein degradation system leads to hyperactivation of ERK signalling which in turn triggers cellular senescence similarly to when driven by oncogenic stress [35, 40, 41]. The accumulation of damaged proteins also increases endoplasmic reticulum (ER) stress, setting off the unfolded protein response (UPS), which then triggers reduced protein synthesis, ER expansion and accelerated protein export [42].

In addition to the macromolecular damage already described, senescent cells also manifest an upregulation of pro‐survival (anti‐apoptotic) pathways and mechanisms aimed at alleviating the impact of DNA and protein damage in the cells [43]. Senescent human fibroblasts are resistant to apoptosis due to persistently elevated levels of anti‐apoptotic BCL2 proteins and the reduced levels of pro‐apoptotic BCL2‐associated protein X (BAX), which may be attributed to senescence‐associated histone modifications [44, 45, 46]. BCL‐W and BCL‐XL, members of the anti‐apoptotic BCL2 family of proteins, are also upregulated in senescent fibroblasts irrespective of the senescence trigger [46]. Moreover, senescence can interfere with apoptosis implementation via downregulation of the main effector caspase‐3 [47]. This senescence‐mediated intrinsic resistance to apoptosis is abrogated upon genetic or pharmacological perturbation of p21^WAF1/CIP1^ or BCL‐2 family members, thus implying the significance of senescence in the promotion of cell survival [46, 48].

#### 1.1.3 Secretory phenotype

The SASP plays an important role in the reinforcement and propagation of senescence phenotypes [49] and contributes to tissue homeostasis [50]. However, it can also have detrimental effects depending on the nature of the triggers, the specific cell types involved and whether senescent cells persist in tissues [51]. The SASP is composed of diverse pro‐inflammatory cytokines, chemokines, growth factors, and matrix metalloproteinases and can function in cell‐autonomous (autocrine) or non‐cell‐autonomous (paracrine) fashions, exerting particular physiological or pathological effects depending on the context [49, 52, 53, 54]. Regulation of the SASP is a complex process that involves different drivers, including nuclear factor kappa light‐chain‐enhancer of activated B cells (NF‐κB) [55], CCAAT/enhancer‐binding protein beta (C/EBPβ) [56], mammalian target of rapamycin (mTOR) [57, 58] and NOTCH1 [59]. CCFs (discussed previously) also activate the cytosolic DNA‐sensing GMP‐AMP synthase stimulator of interferon genes (cGAS‐STING) pathway, an important contributor to innate immunity that participates in driving pro‐inflammatory responses and SASP regulation [29, 60]. The senescence‐associated Alarmin high mobility group box 1 (HMGB1) protein is also implicated in SASP regulation. HMGB1 is exported from the nucleus to the extracellular milieu of senescent cells in a p53‐dependent manner. Depletion of HMGB1 attenuates the secretion of the canonical SASP factor interleukin‐6 (IL‐6) [61]. Intriguingly, the expression levels of HMGB1 were recently proposed to be the key determinant of the fate between senescence and apoptosis in various types of cancer cells following genotoxic stress [62].

#### 1.1.4 Deregulated metabolism

Production of the senescent secretome is highly energy‐demanding, and senescent cells rely heavily on augmented mitochondrial metabolism and glycolysis to meet their ATP needs [63]. Oncogene *BRAF^V600E^*‐induced senescent cells undergo a metabolic reprogramming that is dependent on the mitochondrial gatekeeper pyruvate dehydrogenase (PDH). This process is accompanied by the simultaneous suppression of PDH‐inhibitory enzyme pyruvate dehydrogenase kinase 1 (PDK1) and an increase in the production of the PDH‐activating enzyme pyruvate dehydrogenase phosphatase 2 (PDP2) to promote the use of pyruvate in the tricarboxylic acid cycle, helping senescent cells meet their higher energy requirements [64]. Given the state of perturbed mitochondrial metabolism and its accompanying proteotoxic stress, it is crucial that senescent cells maintain balance between anabolism and catabolism. They do so by a process that couples mTOR to autolysosomes in a distinctive cellular compartment that is known as the TOR‐autophagy spatial coupling compartment, located at the *trans* side of the Golgi apparatus [65]. Currently, an elevated number of lysosomes showing enhanced lysosomal β‐galactosidase activity are the most widely and intensively used marker for the detection of senescence. This can be detected using the senescence‐associated β‐galactosidase (SAβG) assay, where β‐galactosidase activity can be assessed (both *in vitro* and *in vivo*) using a chromogenic reagent at a restrictive pH (pH 6) [66]. Lysosomes in senescent cells also have higher levels of lipofuscin, composed of insoluble lipid‐containing aggregates of lysosomal digestion. Using dyes, these aggregates can also be visualised as an indicator of senescence [67].

Taken together, these hallmarks of senescence discriminate senescent from quiescent or differentiated cells, resulting in their potential utility as biomarkers for senescence detection. Nevertheless, it must be borne in mind that senescence is a highly heterogeneous phenomenon and some of the features discussed above may vary according to different cellular contexts and senescence‐inducing stimuli. Taking into account the complexity and heterogeneity of human tissues in health and disease, the detection of senescent cells in clinical settings requires much more comprehensive studies.

### 1.2 Mechanistic insights into senescence induction in cancer

**Fig. 2:**
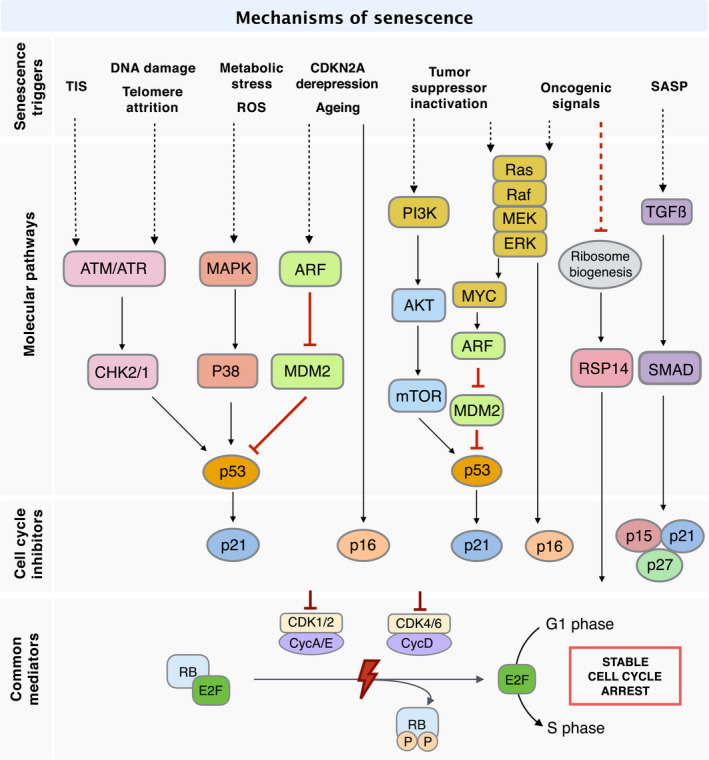
Signalling pathways of senescence induction in cancer. DNA damage and telomere shortening activate a DNA damage response that imposes cell‐cycle arrest through the p53‐p21 axis while ARF and p16 upregulation due to ageing and CDKN2A de‐repression block cell‐cycle progression via both the p53‐p21 and p16 axis. ROS and metabolic alterations implement senescence through MAPK/p38 signalling whereas SASP reinforces senescence by means of TGFβ signalling. Inactivation of tumour suppressors not only induces the Ras/Raf/MEK signalling pathway as oncogenic signals, but also modulates the p53‐p21 axis via the PI3K/AKT/mTOR pathway. In addition to the conventional CKI‐dependent pathway, oncogenic signals trigger cell‐cycle withdrawal by downregulating ribosome biogenesis, thereby increasing RPS14 for direct inhibition of CDK/cyclin‐mediated RB phosphorylation. ATM, ataxia‐telangiectasia mutated; ATR, ATM‐ and Rad3‐related; CDK, cyclin‐dependent kinase; CHK, checkpoint kinase; MAPK, mitogen‐activated protein kinase; MEK, MAPK/ERK kinase; mTOR, mammalian target of rapamycin; PI3K, phosphatidylinositol 3‐kinase; RB, retinoblastoma protein; RPS14, ribosomal protein S14; TGFβ, transforming growth factor β.

While a variety of stimuli may trigger cellular senescence (Fig. 2), DDR induction remains one of the most intensively studied mechanisms [32]. Once sensor protein complexes recognise DNA damage, either apical kinase ataxia‐telangiectasia mutated (ATM) or ATM‐ and Rad3‐related (ATR) is recruited and activated, resulting in the phosphorylation and activation of downstream checkpoint kinases, either CHK1 or CHK2 [68]. Regardless of the specific trigger, DDR signalling cascades ultimately converge on activating the effector protein p53 that subsequently transactivates the CKI p21^WAF1/CIP1^, arresting the cell in G1 or G2/M by blocking activity of CDK2 or CDK1, respectively [69, 70]. In addition to DDR, ageing and epigenetic de‐repression of the *CDKN2A* gene, which encodes the tumour suppressor ARF, also promotes p53‐p21^WAF1/CIP1^‐mediated cellular senescence through the inhibition of MDM2‐mediated p53 degradation [71, 72]. Contrastingly, p16^INK4A^, the other product of the *CDKN2A* gene, interferes directly with cyclin/CDK complexes to impose cell‐cycle arrest [20]. The response to the accumulation of ROS owing to metabolic perturbation includes activation not only of DDR but also mitogen‐activated protein kinase (MAPK)/p38 pathways to promote cellular senescence [73, 74].

The hyperproliferative nature of premalignant cells is associated with the progressive shortening of telomeres [75]. The recognition of shortened telomeres as sites of DNA damage induces replicative senescence via the activation of the DDR signalling cascade (Fig. 2; DNA damage and telomere attrition). It is noteworthy that certain cancer treatments in widespread use effectively implement TIS [76]. Many cancer therapeutics, despite significant mechanistic diversity, induce senescence through the generation of DNA damage, thus eliciting DDR signalling (Fig. 2; TIS).

Senescence can also be triggered in the absence of DNA damage. Since the observation of the first senescence phenotype, in cells ectopically expressing HRas^G12V^, several studies have unravelled the molecular mechanisms underlying OIS and the concomitant accumulation of p53 and p16^INK4A^ [77]. The constitutively active MEK/MAPK cascade upregulates p53 and p16^INK4A^ upon HRas‐induced premature senescence in human fibroblasts [78]. Similarly, Zhu *et al*. [79] proposed that the MEK/MAPK signalling pathway also participates in eliciting senescence in human fibroblasts under conditional activation of Raf, the downstream kinase signal transmitter of Ras. However, they showed that Raf‐induced senescence requires p16^INK4A^, but not p53 and p21^WAF1/CIP1^, thus implying a more nuanced Ras/Raf/MEK/MAPK regulation of OIS [79]. Oncogenic Myc‐induced senescence in B lymphocytes requires both the DDR mediator ATM and p53, highlighting the plausible existence of crosstalk between the DDR and Ras/Raf/MEK/MAPK pathways in OIS regulation [80, 81, 82]. Myc‐induced senescence correlates with delayed lymphoma onset, a process that is suppressed by Cdk2 contributing to lymphomagenesis [83]. Further, a more recent study revealed that Myc promotes OIS through the transactivation of the ubiquitin‐specific protease USP10, which stabilises ARF and thus maintains downstream p53‐mediated senescence [84]. Epigenetic modifications (e.g., H3K9me3 methylation catalysed by the histone methyltransferase Suv39h1), one of the hallmarks of cellular senescence, are involved in Ras‐ or Myc‐driven OIS by suppressing the E2F‐mediated transactivation of proliferative genes [82, 85]. The phosphatidylinositol 3‐kinase (PI3K)/AKT pathway constitutes an additional route to the establishment of OIS since it promotes mTOR‐regulated translation and stabilisation of p53 [86]. In addition to OIS, there are other (indirect) ways to achieve p53‐dependent senescence both *in vivo* and *in vitro*, including the inactivation of other tumour suppressors (TS), such as the phosphatase and tensin homolog (PTEN), an antagonist of the PI3K/AKT pathway [87].

Although the upregulation of CKIs is at the heart of most of the strategies leading to the induction of cellular senescence, a recent study reported a novel mechanism for the induction of senescence that is independent of canonical CKIs. In this study, the accumulation of ribosomal protein S14 (RPS14) arising from reduced ribosome biogenesis triggers senescence through the direct binding of RPS14 to CDK4, thereby inhibiting CDK4 itself as well as RB phosphorylation [88]. Particularly, when we consider that many cancers harbour dysfunctional DDR signalling, this study provides an important insight into the manipulation, and potential redundancy, of senescence pathways in such complex disease settings.

### 1.3 Role of senescence: a double‐edged sword

Although originally viewed as a cellular response to stress and related to ageing and cancer, cellular senescence is also implicated in a plethora of physiological and pathological processes. The ultimate impact of senescence depends on the triggers, stimuli, signalling pathways involved and, crucially, whether senescent cells are efficiently cleared or persist in the tissues [5]. In this regard, transient senescence may play an important role in certain developmental or physiological conditions. More specifically, senescence‐initiated repair processes include SASP‐driven signalling to nearby cells and recruitment of immune cells, clearance of senescent cells by phagocytic cells and activation of nearby stem or progenitor cells to promote repopulation the damaged tissue. These sequential events are brought together in the model of senescence‐clearance‐regeneration [7], where cellular senescence results in a transient (resolved) process that facilitates wound healing [50, 89], limits fibrotic scarring [90] and promotes regeneration [91, 92, 93]. Even during embryonic development, senescence plays a fundamental role in tissue remodelling and organogenesis and facilitates the programmed elimination of transitory embryonic structures and the maintenance of cell balance [94, 95].

However, upon persistent damage or stress, or during ageing, the process of clearance can be compromised by a number of factors, resulting in the accumulation of senescent cells and a chronic inflammatory microenvironment in tissues. The persistence of senescent cells in tissues with age [96, 97] may be also attributed to the decline or exhaustion of immune function, gradually resulting in perturbed tissue homeostasis [7]. As a consequence, senescence is associated with multiple age‐related disorders that include lung fibrosis [98, 99], cardiovascular diseases and atherosclerosis [100, 101], type 1 and 2 diabetes mellitus [102, 103], liver steatosis [104], obesity‐induced metabolic syndrome [105], osteoarthritis [106], sarcopenia [107, 108] and neurological disorders [109, 110, 111]. Accumulation of senescent cells in aged tissues and increased expression of p16^INK4A^ account for the attenuated regenerative functions of stem or progenitor cells [108, 112, 113, 114]. Importantly, in progeroid and naturally aged mice, the selective elimination of senescent cells delays the onset of ageing‐related disorders, resulting in extended median murine life span and health span [107, 115, 116].

Notably, the functions of cellular senescence in cancer development are stage‐ and context‐dependent. Senescence may prevent propagation of premalignant cells by inducing durable cell‐cycle arrest. Conversely, senescence may also promote a tumour‐prone microenvironment and stemness of tumour cells. In Section 2, we will discuss these tumour‐preventing and tumour‐promoting aspects of senescence in more detail.

## 2 Occurrence of senescence in cancer and impact on tumorigenesis

Multicellular eukaryotes have developed mechanisms to counteract the deleterious effects of potentially tumorigenic events – these are the induction of cell death (apoptosis) and a mechanism of permanent cell‐cycle arrest (senescence). Despite these processes sharing an activating mechanism via the DDR‐p53 axis, it is the context that ultimately determines whether cells implement pro‐senescent or pro‐apoptotic programmes [43, 117, 118]. Once activated in premalignant cells, p53 initially transactivates CKI p21^WAF1/CIP1^ inducing cell‐cycle arrest while enhancing the DNA repair response. However, persistent DDR, due to irreparable damage, can result in p53‐mediated transactivation of several pro‐apoptotic genes, including p53 upregulated modulator of apoptosis (PUMA), BAX and BCL2 antagonist/killer (BAK), leading to mitochondria‐mediated apoptotic cell death [119]. However, an alternative mechanism to the elimination of precancerous cells via apoptosis exists, through the p53– p21^WAF1/CIP1^ axis leading to persistent halting of the cell cycle and senescence. This axis also modulates the microenvironment through the SASP, which not only reinforces senescence *in situ*, thus potentially preventing the expansion of precancerous cells, but also promotes immune surveillance to enhance clearance of precancerous cells [51]. Despite both processes appearing vital in tumour suppression, apoptosis and senescence programmes antagonise each other. In apoptotic cancer cells, the p53‐targeted DNA methyltransferase 3a (DNMT3a) represses the senescence programme [120] whereas the levels of anti‐apoptotic BCL‐2 proteins are increased in senescent fibroblasts [44, 45, 46]. We focus here on the induction of cellular senescence in neoplastic growths, both endogenously and as a result of anticancer treatments, and on their respective influences on tumorigenic processes.

### 2.1 Oncogene‐induced senescence in neoplasia

Oncogene‐induced senescence was first observed in cells ectopically expressing *HRAS^G12V^* [77]. This was then followed up by studies revealing the molecular mechanisms underlying OIS implementation, which involve Raf/MEK/MAPK and the downstream effectors p53 and p16^INK4A^ [78, 79]. These results, obtained *in vitro*, laid the initial foundations for the concept of cellular senescence acting as a barrier against oncogene‐driven tumorigenesis before further *in vivo* validation was obtained (Table 1, Mouse models of OIS). The first *in vivo* validation was reported in 20 05 by five independent groups employing different mouse models of OIS [85, 87, 121, 122, 123]. Normal human skin melanocytes bearing the *BRAF^V600E^* mutation acquired a short‐term enhancement in proliferation owing to persistent activation of the Raf/MAPK mitogenic signalling [121]. However, in the longer term, *BRAF^V600E^*‐expressing melanocytes exhibited cell‐cycle arrest with elevated levels of p16^INK4A^ and SAβG activity. Although p16^INK4A^ expression was observed to be heterogeneous among the melanocyte population, the absence of the proliferation marker Ki67 indicated a prevailing growth arrest in human nevi, strongly suggestive of OIS occurrence [121]. Likewise, overexpression of the cell‐cycle related oncogene E2F3 initially promoted cell proliferation resulting in pituitary hyperplasia in mice. Sustained activity of E2F3, however, renders melanotrophs permanently refractory to mitogenic stimulation and induces irreversible cell‐cycle arrest with increased levels of SAHFs and other senescent biomarkers, for example p16^INK4A^ and ARF [123]. Mice bearing a constitutively active *NRas^G12D^* oncogene develop invasive T‐cell lymphomas, usually within one year [85]. The progression of this disease accelerates after selective inactivation of histone methyltransferase Suv39h1, which is required for senescence‐associated H3K9me3 and SAβG activation in response to *NRas^G12D^*. Consistent with the suppression of apoptosis by senescence, the loss of Suv39h1 and therefore of OIS competence renders *NRas*‐driven lymphomas responsive to apoptosis induction [85]. In the case of *KRas^G12V^*‐driven neoplasia in the lung, premalignant lung adenomas exhibited weak proliferation with elevated expression of the senescence biomarkers p16^INK4A^, p15^INK4B^, Dec1, and DcR2 as well as SAβG activity and formation of SAHFs, whereas senescence biomarkers were hardly observed in lung adenocarcinomas, instead staining positive for the proliferative marker Ki67, which implies the presence of OIS in premalignant lesions but not in the established, malignant disease [122]. In a mouse model, the loss of the tumour suppressor PTEN alone promotes development of invasive prostate cancer, whereas the loss of p53 does not [87]. Strikingly, the combined loss of p53, and thereby the competence to induce senescence, and PTEN results in much earlier onset of invasive and highly aggressive prostate cancer. Immunohistochemical analysis confirmed the existence of OIS in *Pten*‐null prostates by the detection of elevated levels of SAβG activity and of expression of ARF, p53 and p21^WAF1/CIP1^, whereas OIS was shown to be absent in double‐null prostates [87]. While preneoplastic cells bearing Ras mutations are more prone to senescence induction, Myc‐driven premalignant cells preferentially favour apoptosis induction [124], suggesting oncogene‐specific effects. However, this is also context‐dependent, as seen in the case of Myc‐driven lymphomagenesis, where DDR mediators and SASP factor TGF‐β1 are both required for senescence induction, suggesting that stroma‐derived pro‐senescence signals play a role in tumorigenesis in this setting [82].

**Table 1:** Incidence of senescence in cancer and premalignancy. 5‐FU, 5‐fluorouracil; γH2AX, phosphorylation of the Ser‐139 residue of the histone variant H2AX; CCL2, C–C‐motif chemokine ligand 2; CDK1, cyclin‐dependent kinase 1; EGFR, epidermal growth factor receptor; ELISA, enzyme‐linked immunosorbent assay; GRO‐alpha/CXCL1, chemokine (C‐X‐C‐motif) ligand 1; HER, human epidermal growth factor receptor; IGFBP‐2, insulin‐like growth factor binding protein 2; IF, immunofluorescence; IHC, immunohistochemistry; IL, interleukin; IR, ionising radiation; NF1, neurofibromin; NSCLC, non‐smallcell lung carcinoma; p53BP1, p53‐binding protein 1; PAI‐1, plasminogen activator inhibitor‐1; PDX, patient‐derived xenografts; Pten, phosphatase and tensin homolog; Rb1, retinoblastoma protein 1; Rheb, Ras homolog enriched in brain; Stat3, signal transducer and activator of transcription 3; TKI, tyrosine kinase inhibitor; VEFGA, vascular endothelial growth factor A.

Gene	Tumour or premalignancy	Mechanism	Reference
**Mouse models of oncogene‐induced senescence**			
*Hras^G12V^*	Breast tumour, bladder tumour, skin papilloma and angiosarcoma	Oncogene activation	[258, 259, 260, 261, 262]
*Kras^G12V^*	Lung adenoma and pancreatic intraductal neoplasia	Oncogene activation	[122]
*Kras^G12D^*	Lung adenoma	Oncogene activation	[263]
*Nras^G12D^*	Lymphoproliferative disorder	Oncogene activation	[85]
*Braf^V600E^*	Nevi, lung adenoma and melanoma	Oncogene activation	[121, 126, 264, 265]
*Rheb*	Prostate intraepithelial neoplasia	Oncogene activation	[266]
*E2f3*	Pituitary hyperplasia	Oncogene activation	[123]
*Akt1*	Prostate intraepithelial neoplasia	Oncogene activation	[267]
*Myc*	Lymphoma, osteosarcoma, liver and lung carcinoma	Oncogene inactivation	[142, 268]
*Trp53*	Sarcoma and liver carcinoma	Tumour suppressor activation	[141, 269]
*Pten*	Prostate intraepithelial neoplasia	Tumour suppressor inactivation	[87, 270]
*Rb1*	Thyroid C cell adenoma	Tumour suppressor inactivation	[271]
*Stat3*	Breast tumour	Tumour suppressor inactivation	[272]
**Human tissues with oncogene‐induced senescence**			
*BRAF^V600E^*	Papillary thyroid carcinomas and nevi	Oncogene activation	[125, 126]
*NF1*	Dermal neurofibromas	Tumour suppressor inactivation	[127]

Therapeutic agent	Tumour or malignancy	Mechanism	Reference
**Therapy‐induced senescence with irradiation (IR) or drugs**			
5‐aza‐2′‐deoxycytidine (Dacogen)	Colorectal tumour, renal cell carcinoma, hepatoma and NSCLC	Inhibition of DNA methyltransferase	[273]
5‐aza‐cytidine (Decitabine)	Malignant pleural mesothelioma	Inhibition of DNA methyltransferase	[274]
Axitinib (Inlyta^®^)	Glioblastoma	TKI	[275]
BRD4770	Pancreatic adenocarcinoma	Inhibition of histone methyltransferase	[276]
Doxorubicin	MMTV‐Wnt1 mice with mammary tumour	DNA damage (DNA intercalator)	[140]
Erlotinib and IR	NSCLC Xenografts of A549 NSCLC	DNA damage and TKI	[129]
Imidazoacridinone C‐1311 (Symadex™)	Colorectal tumour, NSCLC and oesophageal carcinoma	DNA damage (inhibition of topoisomerase II)	[277]
IR	Lung adenoma, breast tumour, colorectal tumour, glioblastoma and neuroblastoma Xenografts of H460 lung carcinoma	DNA damage	[130, 278, 279, 280]
Lapatinib	Breast tumour	HER2‐targeted TKI	[135]
LBH589 (Panobinostat)	Osteosarcoma Xenografts of osteosarcoma	Inhibition of histone deacetylase	[281]
MLN4924	Colorectal tumour, NSCLC, glioblastoma, lymphoma, gastric tumour and osteosarcoma Xenografts of SJSA‐1 osteosarcoma	DNA damage (inhibition of NEDD8 activating enzyme)	[282, 283, 284, 285]
MLN8054	Colorectal tumour and NSCLC Xenografts of HCT116 colorectal tumour	Inhibition of Aurora A kinase	[286]
Neratinib and afatinib	Breast tumour	panHER TKI	[135]
Palbociclib	Patient‐derived sarcoma cells PDX of sarcoma	Inhibition of CDK4/6	[287]
Palbociclib	Melanoma Xenografts of 983B/983BR melanoma	Inhibition of CDK4/6	[288]
Palbociclib and chloroquine or hydroxychloroquine	Breast tumour Xenografts of MCF7‐T breast tumour	Inhibition of CDK4/6 and autophagy	[289]
Ribociclib	Neuroblastoma Xenografts of BE2C, 1643 and EBC1 neuroblastoma	Inhibition of CDK4/6	[290]
Sunitinib (SU11248)	Renal cell carcinoma Xenografts of OS‐RC‐2 renal cell carcinoma	TKI	[137]
Vemurafenib (PLX4032)	Melanoma Xenografts of SK‐MEL‐28 melanoma	Inhibition of BRAF	[291]
VO‐OHpic	MEF and prostate cancer Xenografts of MDA PCa‐2b prostate cancer	Inhibition of PTEN	[292]
WM‐1119	MEF Xenografts of EMRK1184 lymphoma	Inhibition of histone acetyltransferases	[136]
WM‐8014	MEF Zebrafish model of hepatocarcinoma	Inhibition of histone acetyltransferases	[136]

Therapeutic regimens	Malignancy	Evidence of senescence	Reference
**Human tissues with therapy‐induced senescence**			
Neoadjuvant chemotherapy containing cyclophosphamide, doxorubicin, and 5‐FU	Breast cancer	SAβG and IHC of p53 and p21^WAF1/CIP1^	[293]
Neoadjuvant chemotherapy containing carboplatin and docetaxel	NSCLC	SAβG and IHC of CDK1	[185]
Neoadjuvant chemotherapy containing cisplatin and gemcitabine or pemetrexed	Malignant pleural mesothelioma	SAβG and IHC of PAI‐1 and p21^WAF1/CIP1^	[294]
Neoadjuvant chemotherapy containing mitoxantrone	Prostate cancer	qPCR of *p16^INK4a^*, *p21^WAF1/CIP1^* and SASP factors including *IL‐6, IL‐8, GRO‐alpha/CXCL1, IGFBP‐2, and IL‐1beta*	[167]
Neoadjuvant chemotherapy containing sunitinib (SU11248)	Renal cell carcinoma	SAβG and IHC of p53, Dec1, and Ki67	[137]
Neoadjuvant or adjuvant chemotherapy containing anthracycline	Breast cancer	qPCR of *p16^INK4a^* and *ARF*; ELISA of SASP factor VEGFA and CCL2 (assessed using peripheral blood T lymphocytes and patient sera)	[139]
Radiotherapy	Head and neck cancer	IF of γH2AX and p53BP1; IHC of p21^WAF1/CIP1^ (assessed using patient salivary gland)	[177]
Neoadjuvant chemoradiotherapy containing 5‐FU	Rectal cancer	qPCR of *p21^WAF1/CIP1^*, *p16^INK4a^* and *IL‐8*	[138]

Evidence of OIS is also found in human tissues although literature reports are sparse, likely due to the scarcity of available specimens and a paucity of dedicated research. Relevant studies are highlighted in Table 1 (Human tissues with OIS). For example, the expression of the senescence markers SAβG and p16^INK4A^ has been shown to decrease with the increasing malignancy of pigmented lesions. SAβG and p16^INK4A^ were readily detected in benign nevi, but p16^INK4A^ expression was predominantly low in half of dysplastic nevi samples and negative in most areas of invasive melanoma samples [125]. In snap‐frozen tissue sections taken from cases of BRAF^V600E^‐expressing papillary thyroid carcinoma, p16^INK4A^‐positive cells correlated with SAβG‐positive cells, and stable cell‐cycle arrest was further validated by the absence of Ki67 staining [126]. As with senescence induced by inactivation of tumour suppressors, samples from patients with dermal neurofibromas bearing mutated neurofibromin (NF1), which antagonises Ras/MAPK signalling by being a Ras GTPase‐activating protein, tested positive for expression of p16^INK4A^ and for SAβG activity [127].

In summary, the above studies provide the first *in vivo* evidence of OIS and support the notion of cellular senescence being an important tumour suppression mechanism both in mice and in humans [128]. It is crucial that we now enhance our knowledge of OIS in clinical settings, and develop optimised tools for the efficient detection of senescence to facilitate this, including senescence probes (senoprobes) to be used for translational studies in archived and fresh human tissues.

### 2.2 Therapy‐induced senescence in cancer

Cellular senescence takes place not only in the early stages of tumorigenesis but also in more advanced tumours, including in response to DNA damage induced by therapeutic regimens [76]. TIS is in fact a positive outcome of treatment as the proliferation of cancer cells is hampered and immunosurveillance of senescent cells may facilitate clearance of cancer cells, alongside therapy‐induced apoptosis [117]. Radiotherapy, a critically important cancer treatment, is an efficient way of inducing senescence in various p53‐proficient cancer cell types. For instance, 10 Gray radiation doses induce senescence in A549 lung cancer cells [129] and MCF‐7 breast cancer cells, but not in MDA‐MB231 breast cancer cells with hypomorphic p53 [130]. Cell fate decision between radiation‐induced senescence and apoptosis depends in part on the presence of tumour suppressors, as the radiation of PTEN‐deficient human glioma cells induces senescence whereas in PTEN‐proficient cells it triggers apoptosis [131].

A wide number of anticancer drugs with distinct mechanisms of action (many resulting in DNA damage) have been used in *in vitro* and *in vivo* studies of TIS, including bleomycin, cisplatin, cyclophosphamide, docetaxel, doxorubicin, etoposide and palbociclib [8, 132], some of which are also known to induce senescence in clinical settings [Table 1; TIS with irradiation (IR) or drugs]. As it is also the case with therapeutic radiation, the balance between induction of senescence and apoptosis relies heavily on the dose administered. For example, 250 nM of doxorubicin triggers apoptotic cell death of prostate cancer cells [133] while 25 nM of the same drug induces senescence‐like cell‐growth arrest [134]. In addition to TIS caused by DNA‐damaging agents, novel drugs that interfere with oncogenic signalling [e.g., tyrosine kinase inhibitors (TKI)], or epigenetic modifications (e.g., inhibitors of DNA or histone acetyl‐methyltransferase), also contribute to TIS both *in vitro* and *in vivo*. The TKI lapatinib, which targets human epidermal growth factor receptor (HER) family members EGFR and HER2, triggers senescence in breast cancer cells, which showed elevated SAβG activity and expression of the CKIs p15 and p27^KIP1^ following treatment [135]. Also, administration of the small molecule WM‐1119 in lymphoma cells inhibits histone acetyltransferases and induces TIS with upregulated p16^INK4A^ and ARF, leading to lower tumour burden in mouse xenograft experiments [136].

Importantly, increasing numbers of retrospective studies using archived specimens provided by cancer patients who received neoadjuvant or palliative anticancer drugs are being reported (Table 1, Human tissues with TIS). Administration of neoadjuvant sunitinib (a multitargeted TKI) to patients with renal cell carcinoma increases tumoral SAβG activity and senescent biomarkers p53 and DEC1, while downregulating Ki67 [137]. Neoadjuvant treatment of rectal cancer with chemoradiation [including 5‐fluorouracil (5‐FU)] induces senescence with upregulated expression of CKI p21^WAF1/CIP1^ and p16^INK4A^ and of canonical SASP factor IL‐8 at the transcriptional level [138]. Intriguingly, some senescence biomarkers including p16^INK4A^, ARF and SASP components were detected in mRNA extracted from peripheral blood T lymphocytes and patient sera obtained from breast cancer patients treated with chemotherapy. While this likely reflects senescence induction in nontumoral cells, this observation opens up the possibility of assessing TIS using noninvasive methods [139].

In summary, the results described above provide solid evidence for TIS in preclinical and clinical settings and pave the way for studies to fully understand the role of senescent cells in treatment response and ultimately cancer progression. Tools to allow the detection of TIS are crucial for unleashing the clinical utility of TIS determination in prognostic and therapeutic scenarios, including in the detection of cancer recurrence.

### 2.3 Senescence as a tumour‐suppressive response

**Fig. 3:**
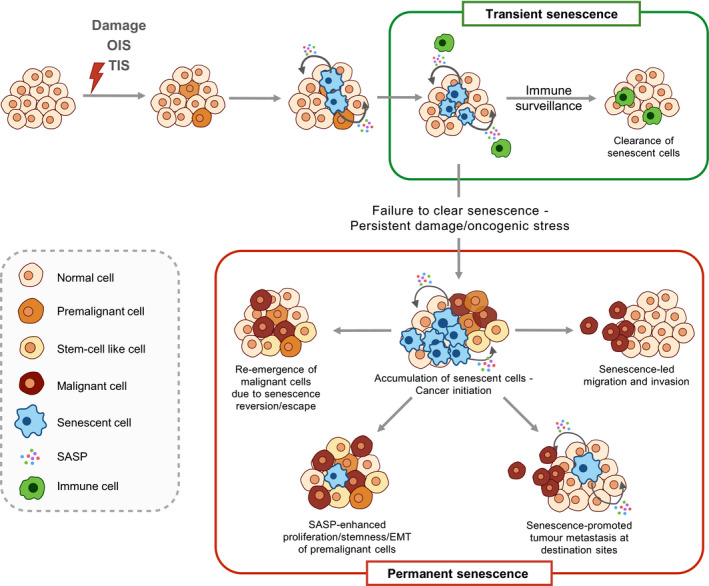
Dual role of senescence in tumorigenesis. Senescence triggered by OIS or TIS initially halts proliferation of premalignant cells and elicits immunosurveillance of senescent cells via SASP secretion, which in turn mediates clearance of premalignant cells, conferring tumour suppression. In contrast, failure to clear senescent cells leads to chronic inflammation by SASP, which cultivates a pro‐tumorigenic microenvironment that promotes proliferation, EMT and stemness of premalignant/malignant cells. Senescence reversion or escape may result in the re‐emergence of malignant cells that may have higher aggressiveness. SASP also contributes to paracrine senescence and induces chemotaxis of malignant cells, resulting in tumour migration, immune evasion and metastasis in distant organs. EMT, epithelial–mesenchymal transition; OIS, oncogene‐induced senescence; SASP, senescence‐associated secretory phenotype; TIS, therapy‐induced senescence.

As described previously, persistent cell‐cycle withdrawal imposed by cellular senescence acts as the first barrier against tumour initiation (Fig. 3, Transient senescence) [85, 87, 121, 122, 123]. Human skin melanocytes bearing the *BRAF^V600E^* mutation exhibit enhanced proliferation in the short term, but in the long term show cell‐cycle arrest as a result of OIS, preventing progression of benign nevi into melanomas [121]. Implementation of OIS as a result of prolonged activity of E2F3 prevents progression of hyperplasia to pituitary tumours, also by inducing irreversible cell‐cycle arrest [123]. The importance of senescence‐mediated cell‐cycle withdrawal in tumour suppression was further highlighted in an *NRas*‐driven lymphoma model, where deficiency of p53 or histone methyltransferase Suv39h1 compromises OIS implementation, leading to lymphomagenesis [85]. The presence of senescence markers in *KRas*‐driven premalignant lung adenomas and their absence in invasive lung adenocarcinomas reflects the importance of OIS in suppressing tumorigenesis [122]. Similarly, the additional loss of *p53* in *Pten*‐null mice, and the consequential compromising of OIS, accelerates the progression of premalignant intraepithelial neoplasias [87].

In addition to providing cell‐cycle arrest as a tumour‐suppressive mechanism, the implementation of cellular senescence also facilitates tumour reversion. Re‐establishment of p53 induces senescence in mouse models of breast cancer and liver carcinoma, resulting in tumour arrest and tumour regression, respectively [140, 141]. In murine models of lymphoma, hepatocellular carcinoma and osteosarcoma, it was observed that cellular senescence programmes remain latently functional in established tumours, and suppression of c‐Myc oncogene‐induced signalling reactivates cellular senescence and promotes tumour regression [142]. Restoration of p53 in Ras‐driven non‐smallcell lung carcinomas decreases the proportion of high‐grade tumours despite failing to induce tumour regression [143]. Interestingly, high‐grade tumours responsive to p53‐mediated tumour arrest show a higher Ras signal as well as ARF expression compared to nonresponsive low‐grade tumours, possibly implying that OIS driven by Ras activation is a prerequisite for efficient tumour arrest by p53 restoration [143].

The SASP, a classic hallmark of senescence, reinforces senescence through secretomes that not only promote the execution of senescence in a cell‐autonomous manner [54, 144], but also contribute to paracrine senescence via IL‐1 signalling and NF‐κB, thereby strengthening the tumour‐suppressive effect [49]. It is worth noting, however, that the senescence‐associated inflammatory response following persistent DNA damage may also result in a form of para‐inflammation that contributes to tumour progression, accelerating growth and invasiveness [145].

Senescence also promotes immunosurveillance for precancerous cells through SASP secretion. Oncogene‐induced senescent hepatocytes promote CD4‐positive T‐cell infiltration and elimination of senescent cells, preventing further advancement of premalignant disease [146]. The association between senescence‐mediated immunosurveillance and tumour suppression was further validated in a model where senescence induction was inhibited by p53 knockdown, resulting in invasive hepatocarcinoma [141]. The restoration of p53 function, and therefore of senescence induction, elicited the infiltration of innate immune cells required for tumour clearance and led to tumour regression [141]. Further evidence in support of a role of SASP in senescence‐mediated tumour suppression was provided using a *NRas*‐driven hepatoma model where blockade of cGAS‐STING‐mediated production of pro‐inflammatory SASP impaired the immunosurveillance‐mediated clearance of *NRasG12V*‐expressing hepatocytes, resulting in intrahepatic tumorigenesis [29]. It is plausible that senescence modulates the tumour‐suppressive immune response via SASP, since senescent human melanocytes upregulate the expression of the major histocompatibility complex (MHC) class II antigen presentation apparatus in response to secreted IL‐1β, thus enhancing T‐cell proliferation *in vivo*, which correlates with better prognosis in melanoma patients [147]. Of note, OIS is accompanied by a dynamic fluctuation of NOTCH1 activity in senescent cells, which can digitally dictate which of two functionally distinct SASP secretomes become predominant [59]. One secretome (first wave) is enriched for TGFβ, contributes to the ‘lateral induction of senescence’ through a juxtacrine NOTCH‐JAG1 pathway and suppresses the senescence‐associated pro‐inflammatory secretome through inhibition of C/EBPβ. The second secretome (second wave) is associated with reduced NOTCH1 activity and involves the upregulation of pro‐inflammatory cytokines, promoting lymphocyte recruitment and senescence surveillance. Interestingly, ligands of the stimulatory natural killer (NK) cell receptor NKG2D were shown to be upregulated in senescent cells irrespective of the trigger, thus increasing NK‐cell‐mediated cytotoxicity towards premalignant cells [148].

In conclusion, cellular senescence is a barrier against tumorigenesis, through halting the proliferation of precancerous/cancer cells, leading to tumour arrest or even regression. SASP‐facilitated paracrine senescence can amplify these tumour‐suppressive effects. The SASP engages in the modulation of senescent cell immunosurveillance, ensuring the clearance of potentially malignant cells. Nevertheless, pro‐inflammatory SASP may be deleterious when the immune system is exhausted, or when senescence is bypassed or compromised (e.g., with the selective inactivation of components required for the implementation of senescence, such as p53, during tumorigenesis), ultimately resulting in cellular escape from senescence‐mediated repression and acquisition of more malignant phenotypes.

### 2.4 Cancer promotion by senescence

Cancer is broadly regarded as a disease of ageing, resulting from the progressive accumulation of damage and stress. Intriguingly, most age‐related stressors, for example DNA damage and replicative exhaustion, induce senescence. In addition to its association with age, cellular senescence can modulate tumorigenesis via the SASP that can nurture chronic inflammation within the tumour microenvironment, which in turn can promote specific aspects of tumour development [149, 150]. Alongside the tumour‐suppressive aspects of senescence described in the preceding section, mounting evidence implicates cellular senescence in tumour progression, including involvement in cancer initiation, promotion, and invasion to metastasis [5, 51]. The impact of cellular senescence on tumour progression can be dissected into proximal effects and distant effects; these will be discussed in the following sections (Fig. 3, Permanent senescence).

#### 2.4.1 Proximal and distant effects of cellular senescence

Since the publication of the first study demonstrating that human fibroblasts undergoing replicative senescence were able to promote the growth of co‐cultured preneoplastic or neoplastic epithelial cells [151], it has been postulated that senescence can manipulate the pro‐tumorigenic microenvironment by means of SASP secretion [51, 52, 152]. Promotion of growth by senescence‐associated secretion can be invoked by multiple senescence triggers, and the stimulating effect on growth observed in preneoplastic epithelial cells can be recapitulated in co‐cultures with senescent cells induced by oxidative stress, oncogenic Ras, or ARF overexpression [151]. Gene expression profiles of senescent human prostate fibroblasts revealed an enrichment of transcripts encoding proteins that promote epithelial proliferation, including the transmembrane glycoprotein amphiregulin, the inhibition of which attenuates the proliferation of epithelial cells in response to conditioned medium (CM) of senescent fibroblasts [153]. With a well‐recognised role in cancer initiation and progression [154], matrix metalloproteinases (MMPs) secreted during senescence facilitate tumour growth in mouse xenografts of breast tumour, which can be abrogated by the broad‐spectrum MMP inhibitor GM6001 [155]. Osteopontin, another SASP factor, stimulates the proliferation of preneoplastic keratinocytes *in vitro* and *in vivo* [156], potentially via the activation of the MAPK signalling pathway [157]. Senescence also appears to account, at least in part, for obesity‐associated cancer development. Dietary/genetic‐induced obesity alters the composition and therefore the metabolites of gut microbiota, one of which is the DNA‐damaging deoxycholic acid [158]. Hepatic stellate cells induce senescence and SASP with abundant pro‐inflammatory factors in response to stimulation with deoxycholic acid. The secreted IL‐1β reinforces the SASP inflammasome, thus promoting the development of hepatocellular carcinoma [158]. Mechanistic studies have further revealed that pro‐tumorigenic SASP is mediated at both the transcriptional and translational level by mTOR, which promotes an IL‐1α/NF‐κB feedback loop for SASP production that led to tumour growth in mouse xenografts of prostate tumour cells [58]. A similar paracrine effect of the SASP was also seen in melanoma, pancreatic cancer and oral squamous cell carcinoma; invasive ability of cancer cells was augmented by CM from senescent fibroblasts, further implicating senescent stromal cells in tumour promotion [159, 160, 161]. Other SASP regulators are beginning to be discovered, for example polypyrimidine tract binding protein 1 (PTBP1) which regulates the pro‐inflammatory SASP by alternative splicing of genes involved in intracellular trafficking [162]. Knockdown of PTBP1 attenuates secretion of pro‐inflammatory factors including IL‐6, IL‐8 and IL‐1α without affecting *NRasG12V*‐driven OIS, thereby impeding tumour growth in mouse livers and in squamous cell carcinoma xenografts. This study identifies SASP inhibition as a promising potential therapeutic strategy against SASP inflammation‐driven cancer [162].

Interestingly, the pro‐tumorigenic effects of the senescent secretome refer not only to increased tumour progression but also to cancer initiation. A recent study demonstrated the potential of the SASP in promoting transformation and tumour initiation of non‐tumorigenic cells [163]. Pituitary embryonic precursor cells can be transformed by expressing mutant oncogenic β‐catenin, resulting in tumours resembling human adamantinomatous craniopharyngioma with clusters of nondividing cells. The presence of markers related to DDR (e.g., γH2AX and phospho‐ATM) and senescence (e.g., p53, p16^INK4A^ and p21^WAF1/CIP1^) within clusters of cells confirmed the implementation of OIS, and the corresponding SASP modulated the pro‐tumorigenic microenvironment. Consistent with the requirement of senescence and SASP in tumour development, precursor cells deficient in the tumour suppressor *Apc* led to reduced OIS and SASP production, as well as to smaller senescent β‐catenin cluster formation, and a mitigated tumorigenic effect [163].

Senescence drives epithelial‐to‐mesenchymal transition (EMT) [164], a cellular transition that helps tumour cells acquire enhanced migratory and invasive abilities [165, 166]. Nonaggressive human breast cancer cells treated with SASP produced by senescent fibroblasts showed reduced cell surface levels of β‐catenin and E‐cadherin, reduced cytokeratin 8/18 expression and increased vimentin expression, all hallmarks of EMT [167]. Further investigation into SASP components revealed that IL‐6 and IL‐8 are major drivers of SASP‐mediated EMT and invasiveness of premalignant or malignant cancer cells, given that their inhibition attenuates the migration into the basement membrane by cells stimulated by senescent CM [167]. Additionally, malignant pleural mesothelioma cells treated with pemetrexed – a standard chemotherapeutic agent used clinically for mesothelioma treatment – undergo accelerated senescence and the addition of senescent CM drives EMT of mesothelioma cells [168]. Pretreatment of mesothelioma cells with senescent CM results in a higher rate of tumour development and earlier onset in mouse xenografts, suggestive of a role for SASP‐mediated EMT in tumour promotion [168]. Similar results were also observed in human colorectal cancer cells upon treatment with senescent CM [138]. Interestingly, in patients receiving neoadjuvant chemoradiotherapy, EMT‐related proteins are upregulated in rectal tumour niches enriched in senescent cells, but not in nearby tumour niches that lack senescent cells, providing *in vivo* evidence for senescence‐associated EMT programming [138].

The increased tumour vascularisation observed when tumorigenic epithelial cells were subcutaneously co‐injected with senescent fibroblasts into mice suggests that senescence may promote tumorigenesis through the stimulation of angiogenesis [169]. A similar angiogenic effect was also reported in hypoxia‐induced senescence in mouse retina cells, where the senescent secretome contributed to pathological retinopathic angiogenesis [170]. SASP factors secreted by senescent (owing to aneuploidy‐related chromosomal instability) retinal pigment epithelial cells confer angiogenic capabilities, as the CM collected from cultured senescent cells promotes the vascular sprouting of mouse choroid explants [171]. Moreover, highly aneuploid and senescent cells are located at the invasive edge of the tumour in samples from patients with invasive ductal breast carcinoma [171]. Interestingly, vascular endothelial growth factor (VEGF) is only partly responsible for promoting angiogenesis, as the senescent CM pretreated with VEGF‐neutralising antibody was unable to completely block the invasion of endothelial cells into the basement membrane, implying the presence of other angiogenic factors in the SASP [169].

In contrast to senescence‐mediated immunosurveillance of precancerous cells, an age‐related accumulation of p16^INK4A^‐positive senescent T cells, which is implicated in negative modulation of the adaptive immune response, may be responsible for pro‐tumorigenic effects through reduced immune clearance of premalignant cells [97, 172]. Indeed, lineage‐specific deletion of p16^INK4A^ can rescue severe age‐related functional decline of T cells, thus facilitating homeostatic proliferation and antigen‐specific immune responses [173]. Although senescent stroma can induce infiltration of immune cells via a pro‐inflammatory SASP, the increase in myeloid cells and the concurrent decrease in lymphocytes within the infiltrating population are indicative of an immunosuppressive microenvironment that may correlate with potentially limited immunosurveillance [174]. Accordingly, cancer cells co‐injected with senescent fibroblasts into immune‐competent mice induced larger tumours volumes than those co‐injected with nonsenescent fibroblasts, whereas co‐injection in immunocompromised nude mice led to equivalent tumour growth irrespective of fibroblast status [174]. In addition, immunosuppression and therefore evasion of senescent cells can be accomplished through the pro‐inflammatory SASP factor IL‐6, which induces the expression of MHC molecule HLA‐E [175]. Once expressed on the surface of senescent cells, HLA‐E interacts with inhibitory receptors on NK cells and CD8‐positive T cells, resulting in the evasion of senescent cells from immune surveillance [175]. As the tumour‐suppressive effects of senescence appear to rely heavily on immune surveillance, the decline of immune cytotoxicity of senescent cells leads to increased senescent cell accumulation and chronic inflammation (Fig. 3, Permanent senescence), which not only correlates with poor health span and shorter life span, but also fosters a pro‐tumorigenic microenvironment [176].

The systemic impact of cancer therapies is evidenced by the increase in the levels of senescence markers and functional decline in noncancerous tissues after radiation or chemotherapy. For instance, the blood of patients after chemotherapy shows higher amounts of CD3‐positive lymphocytes expressing p16^INK4a^ [139], and patients with head and neck cancer who received radiotherapy are predisposed in an ‘in field’ and senescence‐driven loss of salivary gland function [177]. Cellular senescence may also therefore be able to adapt the distant microenvironment to affect tumour invasion and metastasis, in addition to its capacity to modulate the proximal microenvironment in tumorigenesis. In line with the systemic effects of senescence observed in cancer patients, SASP factors including IL‐11 and angiopoietin‐like 4 were detected in the plasma of mice engrafted with HER2‐driven senescent tumour cells, contributing to larger metastases from proliferating tumour cells [178]. Consistent with the known increase of the senescence burden with age, the abundance of SASP factor Chemerin is higher in skin dermal fibroblasts from older donors than from young ones [179]. *In vitro* studies with Chemerin revealed that it increases the migratory ability of cutaneous squamous cell carcinoma cell lines. Such augmented migration promoted by the senescent stroma can be attributed not only to SASP‐mediated chemotaxis [179] but also to SASP‐induced reorganisation of actin and microtubule cytoskeleton networks, leading to reduced focal adhesion and traction forces, thus endowing cancer cells with more aggressive migratory behaviours [180]. In addition to the influence exerted by the senescent stroma, senescent cells within tumour clusters can also function as navigators for tumour invasion, being strongly implicated in the collective invasion and metastasis in tissues from patients with *BRAF^V600E^*‐expressing papillary thyroid carcinoma [126]. Moreover, senescent tumour cells were found as aggregating centres for formation of three‐dimensional tumour clusters in densely plated (monolayer) MDA‐MB‐231 breast cancer cells [181]. Despite comprising only a small portion of the total isolated primary tumour cells, senescent tumour cells exhibited higher migration ability than nonsenescent tumour cells [126]. Further *in vivo* investigation unravelled that senescent tumour cells generated a gradient of C‐X‐C‐motif ligand (CXCL)12, a SASP factor, in the invasive region of the tumours, orchestrating the collective invasion so that senescent cells led the primary invasion followed by nonsenescent cells. Genetic perturbation of the CXCL12 gradient not only markedly attenuated tumour invasion, but also abrogated anoikis resistance, highlighting the significance of SASP in mediating tumour invasion and potential metastasis [126].

Considering that metastatic bone lesions are prevalent in patients with advanced breast cancer, it was proposed that senescent osteoblasts (which may be induced by treatment) increase local osteoclastogenesis via SASP factor IL‐6 and nourish a pro‐metastatic niche for subsequent seeding and outgrowth of breast cancer cells [182]. Management of the primary breast tumour by systemic doxorubicin treatment is overcome by tumour resistance within weeks, with subsequent metastases in the liver and lung. However, elimination of p16^INK4a^‐positive senescent nontumour cells in hosts resulted in significantly fewer metastases [183]. In an attempt to simulate a standard paradigm of breast cancer treatment, researchers surgically removed breast tumours prior to administering doxorubicin treatment. After a short latency, primary tumours recurred in all mice, although mice in which the senescent cells had been eliminated showed smaller recurring tumours (in size) and fewer metastases. This dramatic improvement suggests that chemotherapy can promote tumour growth and metastasis by inducing senescence in nontumour cells [183].

In summary, senescent cells modulate neighbouring preneoplastic cells via SASP, which promotes cancer initiation, proliferation and progression. Senescence induces EMT of malignant cells that enables enhanced migratory and invasive abilities. To support tumour growth, SASP factors including VEGF also stimulate angiogenesis. Importantly, senescent cells attenuate immunosurveillance for premalignant cells either through cultivating an immunosuppressive microenvironment or through reducing immune cytotoxicity of senescent cells, facilitating tumour progression. The above studies also provide evidence for the role of senescence in facilitating tumour metastasis, which is accomplished either by promoting the migratory ability of tumours *in situ* or by preparing the microenvironment in the distant organ for tumour seeding. While these factors complicate our understanding of TIS, they are crucial for understanding systemic contributions of senescence to cancer, with important clinical implications for both local and distant tumour control.

### 2.5 Tumour progression facilitated by senescence reversion

The well‐established concept of irreversible senescence in normal cells [184] has been challenged by studies showing that therapy‐induced senescent cancer cells can escape the imposed cell‐cycle arrest and resume cell‐cycle progression. In a pioneering work using human non‐smallcell lung cancer (NSCLC) H1299 cell lines deficient in p53 and p16^INK4A^ but with a competent RB pathway, senescence induction in response to camptothecin was proposed to be reversed in minority cells after extended observation. This was rare, occurring in ~ 1 in 10^6^ cells, and was dependent on the increased expression of CDC2/CDK1 protein in senescence‐escaping cells [185]. A recent study performed using a B‐cell lymphoma mouse model further uncovered the potential pro‐tumorigenic threat posed by these previously senescent cells. Adriamycin (doxorubicin)‐induced senescent cancer cells acquired gene expression patterns similar to those of adult tissue stem cells, which then re‐entered the cell cycle and exhibited a higher tumour‐promoting capacity [186]. However, this process was shown using an experimentally induced exit from TIS, through genetic manipulation to reverse senescence‐associated histone methylation (and its related epigenetic changes). Lineage tracing of senescent H460 human lung cancer cells after etoposide treatment confirmed that a few cells with senescence phenotypes regained their ability to divide while the majority of senescent cells remained under persistent growth arrest [187]. The re‐emergent dividing cells also acquired stem cell‐like self‐renewal capacity and led to tumour formation when injected into immunodeficient mice. Similar observations were obtained when doxorubicin‐induced senescent 4T1 breast cancer cells were implanted into the mammary fat pad of both immunocompetent and immunodeficient mice, indicating the *in vivo* tumorigenic capability of senescent cells [187]. The mechanism underlying senescence reversion remains to be elucidated, although a recent study proposed that the SASP factor thrombospondin‐1 and its receptor CD47 may play key roles in preventing senescence escape of cancer cells upon TIS [188]. This raises the possibility that ‘senescence reversion’ observed for therapy‐induced senescent cancer cells might instead reflect ‘senescence escape’, a property that might be acquired during the previous and sequential steps required for the full implementation of the senescence programme – that is, these cells never become fully senescent. Therefore, such (pre)senescent cells might be more likely to re‐enter the cell cycle than *bona fide* (fully) senescent cells, which require the successful completion of a number of genetic and epigenetic alterations [4]. Whether senescence reversion and/or escape occur in other biological contexts and clinical settings still requires formal demonstration.

Regardless of the mechanisms by which such cells regain the capacity to divide, previously senescent cancer cells may acquire their aggressiveness through the process of senescence entry and exit, after which they then exhibit enhanced migratory and invasive behaviour [189]. Previous studies have demonstrated that damage‐ or ageing‐induced senescence may precondition the microenvironment for cell reprogramming, in part through secretion IL‐6 [190, 191], whereas OIS promotes the regenerative competence of primary mouse keratinocytes via upregulating transcripts associated with somatic and cancer stem cells [192]. In multiple myeloma and human kidney premalignant cells, the SASP arising upon TIS or OIS might drive the emergence of cancer stem‐like cells (CSLCs), thus promoting tumorigenesis and cancer progression [193, 194]. Senescence induction of p53‐competent nonstem leukaemia cells by adriamycin treatment leads to increased expression of leukaemia stem cell surface markers and upregulation of stem cell‐related transcripts, which was minimal in their p53‐deficient nonsenescent counterparts [186]. After genetic reversion of senescence by p53 inactivation, these previously senescent cells prompted leukaemia initiation while cells that had never experienced senescence rarely induced leukaemia in the recipient mice, implying the pro‐tumorigenic potential of senescence‐associated cell reprogramming [186]. Nevertheless, more research using refined lineage tracing of senescent cells (in diverse cancer types) is required to verify the association of senescence‐mediated reprogramming with acquired stemness of cancer cells.

In summary, although cellular senescence is implemented as a barrier to early tumorigenesis and is also induced as positive outcome in the initial response to cancer therapy, the inefficient clearance of senescent cells by immunosurveillance can result in persistent senescence. SASP‐mediated chronic inflammation within the tumour microenvironment favours tumour initiation, progression, angiogenesis, invasion and migration. Senescence and accompanying SASP also engage in modulating niche locations in distal organs to promote tumour metastasis. Furthermore, emerging evidence suggests that a small number of cancer cells can be made senescent by cytotoxic therapies but then revert to active proliferation. While this phenomenon of senescence reversion or escape remains the subject of intense debate, we must urgently and intensively study this possibility given the stem cell‐like and aggressive features that have been shown already and the associated implications for tumour progression/recurrence and metastatic spread. Better tools for detecting senescent cells *in vivo* will be crucial to elucidate the translational importance of each of these aspects.

## 3 Approaches for the detection of senescence in cancer

Given that no single universal senescence marker has been identified to date [11, 24, 195] detection of senescent cells in tissues is conventionally attained by using a battery of immunohistochemical approaches [196] to probe for the presence of molecular biomarkers involved in signalling pathways specific for tumour suppression (e.g., p53 and RB) or cell‐cycle arrest (e.g., p16^INKA^ and p21^WAF1/CIP1^), senescence‐associated epigenetic changes (e.g., SAHFs and H3K9me3), lack of proliferative capacity assessed by monitoring the incorporation of nucleoside analogues [e.g., 5‐bromo‐2′‐deoxyuridine (BrdU) or 5‐ethynyl‐2′‐deoxyuridine (EdU)] into newly synthesised DNA, together with the detection of elevated SAβG activity [197]. In addition to their intrinsic specificity issues, the conventional methods also require fresh or deep‐frozen tissues, which further restrict their use in *in vivo* settings for real‐time senescence detection.

**Fig. 4:**
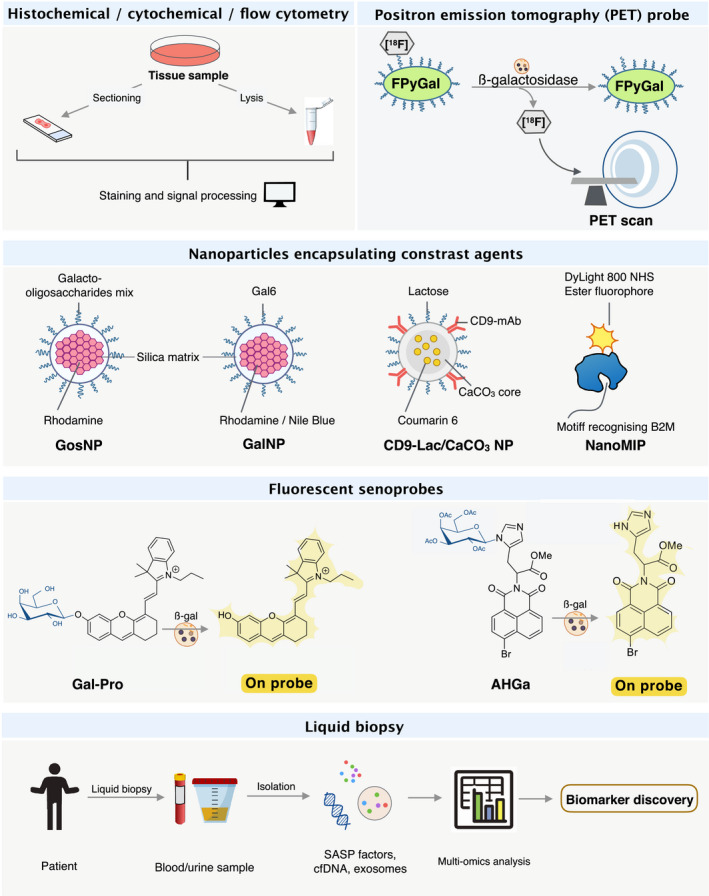
Novel approaches for *in vivo* senescence detection. In addition to conventional detection methods relying on IHC detection of multiple senescence biomarkers in deep‐frozen or fixed tissues, recent development of approaches combining histochemical, cytochemical and flow cytometry offer higher efficiency for senescence detection in fresh tissues. The fine tuning of nanoparticles for recognising senescent cells strengthens further the targeted delivery of cargoes, that is, image contrasting agents, into senescent tumour cells. Avoiding potential cytotoxicity, OFF‐ON Senoprobes facilitate the real‐time detection and tracking of living senescent cells with elevated SAβG activity. In the human setting, the senescent‐specific PET probe FPyGal may be used to assess senescence burden within tumours pre‐ and post‐treatment, which would provide valuable information in the design of therapeutic strategies and inpatient response. The emerging field of cell‐free DNA (cfDNA) analysis in liquid biopsy provides the least invasive senescence detection tool that is also usable in large‐scale and longitudinal patient screening and monitoring. B2M, β_2_ microglobulin; nanoMIP, molecular imprinted nanoparticle; NP, nanoparticle; SAβG, senescence‐associated β‐galactosidase.

Bearing in mind that even a low senescence burden may contribute to tumour suppression or, when pathological, exacerbate tumour progression and facilitate relapse, it is important that we develop tools to accurately, and sensitively, identify and track senescent cells *in vivo*. Tracking senescent cells *in vivo* may help to identify the presence of premalignant lesions attributed to OIS as well as to establish the distribution of senescent cells within these lesions, which can indicate of the potential of tumour progression (prognosis) and be used as an additional tool to facilitate patient stratification and early (preventative) intervention. Additionally, *in vivo* detection of senescent cells can be applied to assess patient response to radiotherapy and chemotherapy interventions, where TIS occurrence may be considered for devising specific therapeutic strategies and proactive follow‐up. Last but not least, tracking senescent cells will be pivotal for post‐treatment assessment since the presence of TIS cells may pose a potential risk of recurrence. Although TIS‐focused cancer therapies have been widely proposed [198, 199], their potential efficacy *in vivo* will require acute assessment of the response coupled to the precise tracking of senescent cells. We discuss here some recent inventions designed to provide precise and real‐time tracking of senescent cells in preclinical models with potential clinical applications (see Fig. 4 and Table 2).

**Table 2:** Summary of novel & potential inventions for senescence detection in cancer. γH2AX, phosphorylation of the Ser‐139 residue of the histone variant H2AX; B2M, β_2_ microglobulin; HGMB1, high mobility group box‐1; nanoMIP, molecularly imprinted nanoparticles; NB, Nile blue; NIR, near‐infrared; NP, nanoparticle; PET, positron emission tomography; SAβG, senescence‐associated β‐galactosidase; SBB, Sudan Black B.

Type	Markers	Method of detection	Application
Histochemical, cytochemical and flow cytometry	SAβG activity Ki67, HGMB1, γH2AX	ImageStreamX (flow cytometry and image analysis)	*In vitro* & *in vivo* [201]
	Lipofuscin accumulation	SBB with biotin conjugation ‐ GL13	*In vitro* & *in vivo* [67]
Senoprobe	SAβG activity	NIR fluorescent probes ‐ Gal‐Pro ‐ NIR‐BG	*In vitro* [220] *In vivo* [221]
	SAβG activity	Two‐photon fluorescence probe ‐ SG1 ‐ AHGa	*In vitro* [223] *In vitro* & *in vivo* [224]
Nanoparticle	SAβG activity	GosNPs	*In vitro* [210]
	SAβG activity	GalNPs	*In vitro* & *in vivo* [99]
	SAβG activity	S3 (GalNPs with NIR dye NB)	*In vitro* & *in vivo* [211]
	SAβG activity CD9 receptor	CD9‐Lac/CaCO_3_ NPs	*In vitro* [215]
	B2M epitope	B2M nanoMIPs	*In vitro* & *in vivo* [216]
PET probe	SAβG activity	PET tracer – [^18^F]FPyGal	*In vitro* & *in vivo* First‐in‐man [229]
Liquid biopsy	DNA methylation	Genome‐wide DNA methylation analysis	*In vitro* & *in vivo* [235] *In vitro* & *in vivo* (human samples) [236]

### 3.1 Senescence detection: histochemical, cytochemical and flow cytometry

In addition to SAβG activity, used widely in senescence‐detecting assays in tissues [197], the pigment granule lipofuscin is another potential candidate for assay development. Lipofuscin accumulates upon senescence induction and consists of lipid‐containing residua of lysosomal digestion. The granules can be readily detected by staining with Sudan Black B (SBB) dye [200]. The heterogeneity in granule size and background noise (partially owing to impurities during commercial manufacturing) compromises the diagnostic power of SBB. This led to the development of a *de novo* synthesised compound structurally simulating SBB and employing biotin conjugation – GL13 – which can be used for signal amplification by an immunohistochemical–enzymatic reaction. Following a two‐step staining procedure, it was determined that the biotinylated compound stains senescent cells with an improved sensitivity and increased signal‐to‐noise ratio across a range of biological materials from cultured cells, fresh/frozen tissues, to formalin‐fixed and paraffin‐embedded (FFPE) tissues, the latter importantly broadening its use to archived clinical samples [67].

Conventional identification of senescent cells requires immunohistochemical staining of multiple senescence biomarkers to prevent false positives seen when senescence is assumed on the basis of a single positive marker. Current protocols for the assessment of SAβG activity, however, do not usually support the simultaneous detection of additional senescence biomarkers or any other co‐stains to reliably identify cell types. Precise quantification of senescent cells within tissues would facilitate a better understanding of how senescence is involved in cell and tissue biology in different contexts, but it is not possible with currently available approaches. In an attempt to circumvent these issues, the identification of senescent cells on a single‐cell basis was pursued using flow cytometry techniques combined with high‐resolution image analysis where SAβG assays are performed in parallel to the determination of cell type‐specific markers or other senescence‐related markers such as HGMB1, γH2AX and absence of Ki67. This method allows a more accurate annotation of cellular states in certain cell types and detects senescent cells in murine tumours and fibrotic tissues. Also, this approach is quantitative, allowing larger‐sized senescent cells and normal cells to be distinguished *in vivo* [201].

The combination of flow cytometry techniques with histochemical or cytochemical approaches strengthens the accurate detection and quantification of senescent cells *in vivo*. However, this requires physical tissue collection, which may limit the application of this combination approach. Tools that allow *in situ* detection of senescent cells would allow broader research and clinical utility.

### 3.2 Nanoparticles targeting senescence

In the pursuit of precision cancer diagnostics and therapy, nanoparticles have emerged as versatile candidates with incredibly broad potentials for clinical application [202, 203, 204]. Nanoparticles, defined as any material in which at least one of its dimensions ranges between 1 and 100 nm, possess distinct properties from their bulk counterparts that allow for widespread application across diverse fields, including the medical application of smaller, inorganic‐based nanoparticles which have had significant recent interest [205, 206, 207]. With a core‐shell (or multiple shell) structure, inorganic nanoparticles function as a degradable gated system to protect their encapsulated cargoes and to release them deliberately when appropriate stimuli are detected [208]. Given that such controllable release can be achieved using biodegradable nanoparticles [209], the characteristically elevated levels of SAβG activity in senescent cells have become an exciting feature to be exploited for targeted delivery of drugs and detection probes. Of relevance to this field, the first senescence‐targeted mesoporous silica‐based nanoparticles were manufactured endowed with a galacto‐oligosaccharide‐coated shell (GosNPs), which is a substrate of SAβG. Once these nanoparticles are endocytosed by the cells and fused with lysosomes, SAβG‐mediated hydrolysis of galacto‐oligosaccharides enables the release of an encapsulated fluorescent dye (rhodamine) in human senescent fibroblasts but not in nonsenescent cells [210]. The delivery system was improved further by capping silica‐based nanoparticles homogeneously with 6‐mer galacto‐oligosaccharides (GalNPs) since this enhances the specificity of SAβG‐mediated release of cargoes. This approach was validated in mouse xenografts of SK‐MEL‐103 melanoma and NCI‐H226 non‐small‐cell lung cancer (NSCLC) cells, where GalNPs released the encapsulated fluorophore in palbociclib‐treated senescent tumour xenografts but not in untreated tumour xenografts, providing the first *in vivo* proof of principle of senescence detection and therapeutic targeting using nanoparticles [99]. The detection power of GalNPs was recently further improved with the combined use of a near‐infrared (NIR) dye Nile blue (NB), termed S3 [211]. Mesoporous silica GalNPs have a high loading capacity so that NB can be entrapped at high concentration for optimal emission quenching. As a result, S3 administration to mice bearing breast tumours elicited only negligible fluorescence signals while its administration to breast tumour xenografts subjected to TIS by palbociclib treatment contributed to sharp fluorescence signals, showing efficient quenching and specific release of NB in senescent cells that had increased SAβG activity [211].

Despite the preferential release of cargoes in senescent cells compared with nonsenescent cells, the uptake of GosNPs and GalNPs is ubiquitous, which poses a threat of (cargo and coat‐dependent) cytotoxicity in normal cells, important when considering potential clinical applications. While nanoparticles may have some passive accumulation in tumour tissues owing to the enhanced permeability and retention (EPR) effect from often dysfunctional vascular and lymphatic systems [212, 213], in compilation studies derived from 232 datasets, despite some preferential retention of nanoparticles in tumours via EPR, only 0.7% (median) of administered nanoparticles reached tumours [214]. This effect remains highly controversial in humans. Consequently, the additional advantage of bioengineered and functionalised nanoparticles can be exploited by targeting cell surface receptors overexpressed on specific or particular cell types, in order to enhance preferential uptake by certain cells. Accordingly, and given that CD9 is overexpressed in senescent cells, researchers conjugated calcium carbonate nanoparticles with CD9 monoclonal antibody, followed by wrapping the nanoparticles with a lactose‐polyethylene glycol conjugate for optimised stabilisation in the blood and prevention of opsonisation (CD9‐Lac/CaCO_3_ NPs). CD9‐Lac/CaCO_3_ NPs were specifically taken up by senescent cells via interaction with CD9 receptors, before SAβG‐mediated release of cargoes occurred intracellularly, as evidenced by the presence of encapsulated fluorescent probe Coumarin 6 in senescent primary human dermal fibroblasts but not in their nonsenescent counterparts [215]. A recent study using molecularly imprinted nanoparticles (nanoMIPs) to target an extracellular component of MHC I molecules – β_2_ microglobulin (B2M) – allowed selective detection of senescent cells induced by overexpressing p16^INK4A^ [216]. When intravenously injected, B2M nanoMIPs tagged with DyLight 800 NHS Ester showed fluorescent signals in elder but not in young mice, at least partially reflecting the higher number of senescent cells present in aged tissues. However, the preferential accumulation of B2M nanoMIPs in the gastrointestinal tract rather than in other organs might hamper its application in whole‐body senescence detection [216].

The development of senescent cell‐targeted nanoparticles capable of encapsulating various types of contents extends their potential use in the clinical setting, and may allow both drug and imaging contrast delivery. The low delivery rate of administered nanoparticles to tumours and potential toxicities might, however, reduce their therapeutic windows [204].

### 3.3 Fluorescent senoprobes

The development of OFF‐ON fluorescent senoprobes has drawn considerable attention in recent years. As the presence of elevated numbers of lysosomes is an important hallmark of senescence [11], the accompanying high‐level SAβG activity has garnered significant attention in the development of detection assays based on this lysosomal enzymatic activity. Building on traditional SAβG assays relying on chromogenic changes upon hydrolysis of its substrates, a wide number of chromogenic or fluorogenic probes are now available for the detection of senescent cells [217]. However, drawbacks such as low tissue penetrance and autofluorescence from specimens may hamper the *in vivo* use of commercially available fluorescent senoprobes such as SPiDER‐βGal [218]. Although some NIR probes have been designed to circumvent the issue of penetrance, they were validated using cells expressing ectopic β‐galactosidase via *LacZ* gene transfection instead of endogenous SAβG [219]. The recent design of OFF‐ON NIR fluorescent probes – Gal‐Pro [220] and NIR‐BG [221] – remarkably strengthen our ability to detect (in real time) senescent cells, based on the endogenous (lysosomal) SAβG activity in *in vitro* and *in vivo* settings, respectively. The feature of Gal‐Pro anchoring to intracellular proteins to promote its accumulation makes it possible to achieve single‐cell resolution when using these probes in living cells [220] while the longer emission wavelengths (708 nm) of NIR‐BG further overcome limits for deep‐tissue imaging, making the first NIR imaging of genuine SAβG activity in xenografts of human tumours in mice possible [221]. S3 (discussed above), which is composed of GalNPs and the NIR dye Nile blue, quenches autofluorescence by packing NB at high concentration in GalNPs, ensuring signals are detected in senescent cells only and with negligible background [211].

The development of two‐photon microscopy has further circumvented issues with the light‐scattering nature of most biological tissues, allowing high‐resolution deep imaging in organs of animals [222]. The combined use of two‐photon microscopy with the ratiometric two‐photon fluorescence probe SG1, which produces intensified emission upon reaction with SAβG, provides a more precise and quantitative detection of senescent cells in rat skin tissues [223]. Taking advantage of the deeper tissue penetrance offered by the two‐photon fluorescence technology, the OFF‐ON probe AHGa accomplished the first *in vivo* detection of senescent cells in a model of palbociclib‐treated tumour xenografts, which can be transformed into AH by SAβG in senescent cells, resulting in intense fluorescence signals for visualisation [224].

The OFF‐ON NIR and two‐photon fluorescent probes enable the precision detection and tracking of senescent cells within whole animals without raising significant cytotoxicity concerns. When it comes to human use, however, the utility for whole‐body screening using these senoprobes remains questionable. In this regard, the design of probes based on currently available whole‐body imaging technologies would therefore undoubtedly be a great advance.

### 3.4 Positron emission tomography senoprobes

Nuclear medicine allows to couple noninvasive imaging techniques to small amounts of radioactive materials (or radiopharmaceuticals) that can target tumours or even the tumour microenvironment [225]. Positron emission tomography (PET) imaging is a potent tool for collecting information about physiological, anatomic and biochemical properties of cancer cells [226]. In contrast to the aforementioned detection approaches, PET has already been widely used in clinical settings for cancer detection and staging and for assessing therapeutic response [227, 228]. Radioactive tracers are required for PET imaging – these can be designed for visualising biochemical changes, including SAβG activity within senescent cells. A novel β‐galactosidase specific PET tracer – [^18^F]FPyGal – and the first‐in‐man study was reported [229]. The specific uptake of FPyGal by senescent cells was first validated in *in vitro* models and subsequently applied to mouse tumour models subjected to senescence induction. In this study, consistent results were observed showing correlation of lysosomal β‐galactosidase activity and FPyGal uptake in tumours. A case study in a cancer patient treated with the anticancer agent alisertib further revealed a high uptake of FPyGal in a liver metastasis [229]. In addition to enzymatic changes, to enable more precise detection the radioactive tracers can be designed to target specific proteins/pathways related to cellular senescence (e.g., p16^INKA^ or p53 in DDR). As far as we are aware, such probes targeting senescent biomarkers have not yet been developed, although some proof‐of‐concept studies tracking immune cells *in vivo* using anti‐CD8 PET probes are available [230, 231].

Detection of senescent cells using PET probes would provide a noninvasive approach for the detection of OIS and TIS, alongside conventional tumour cross‐sectional imaging. There may be some concerns about the use of radioactive materials required in PET imaging, but these risks are generally low.

### 3.5 Senescence detection in liquid biopsies

Liquid biopsies approaches have emerged in recent years, allowing longitudinal assessment of tumour burden and subclones in a minimally invasive manner [232]. The analysis of cell‐free DNA (cfDNA), which may derive from either cancer cells or cells within the tumour microenvironment, holds promise for unravelling cancer‐associated genetic or epigenetic alterations that can in turn be used as biomarkers for early cancer detection, prognosis and monitoring [233]. In the context of early cancer detection, cfDNA analysis is a technique possessing sufficient specificity and sensitivity to discriminate signs of genomic alterations between cancer patients and healthy individuals [234]. Considering that cellular senescence is commonly a response to DNA damage and stress, which implies fundamental epigenetic changes [21, 22], it is tempting to speculate that DNA methylation signatures determined via cfDNA analysis could indicate the presence of senescence phenotypes and therefore potentially suitable for longitudinal monitoring of senescent cell burdens [235, 236]. Senescent cells generally exhibit hypermethylation of promoters associated with metabolic regulators, while transformed cells exhibit hypermethylation of promoters involved in survival and developmental genes [235]. *HRas*‐driven OIS, however, is associated with minimal changes in DNA methylation compared with replicative cellular senescence or early transformed cells [235]. DNAmSen is a predictor of cellular senescence that is related to three distinct senescence inducers (replicative stress, OIS and IR), developed through analysis of DNA methylation patterns that distinguish between early passage cells and senescent cells [236]. DNAmSen values correlate not only with age, reflecting a senescence burden that is higher in elder donors than in young ones, but also with the severity of age‐related diseases such as lung tumours [236]. In combination with a human tissue‐specific or cell type‐specific methylation atlas, cfDNA analysis may provide information to map senescence to a specific organ or cell type [237, 238], although there is recent evidence that cellular senescence may also block irradiation‐induced cfDNA release [239].

In addition to the direct capture of cfDNA in peripheral blood, secreted extracellular vesicles (EVs) present in liquid biopsies are another potential liquid biopsy target worth exploring [232]. EVs contain numerous molecules including proteins, lipids, RNA and DNA and may represent a source of senescence and cell type‐specific biomarkers [240]. Whole‐genome sequencing of DNA in EV isolated from plasma of patients with prostate cancer revealed genetic perturbations of senescence‐associated oncogenic signalling pathways, for example Myc, AKT and PTEN [241], implying the potential of interrogating EV contents for senescence detection. In addition to EVs, circulating SASP factors may also potentially be used to indirectly assess cellular senescence in tissues [242, 243]. Diligent assessment of circulating SASP factors showed that soluble and EV‐carried SASP proteomes correlate with human plasma ageing markers [242]. Also, the plasma concentrations of seven circulating SASP factors are highly correlated with biological age and adverse clinical outcomes [243]. Considering the abundance and heterogeneity of SASP cocktails secreted by senescent cells, advanced technologies capturing alterations of the senescent secretomes may allow detection of senescence phenotypes in liquid biopsy with increased precision.

Although the detection of senescent cells via liquid biopsy remains in its infancy, it is an exciting prospect that is worthy of further exploration in translational clinical studies in cancer patients.

## 4 Future perspectives and challenges

Cellular senescence is a response to stress and oncogene activation that promotes repair, contributes to tissues homeostasis and protects us from cancer by inducing a stable cell‐cycle arrest and imposing a complex secretory phenotype that affects the nearby tissue. The senescence programme is therefore presumably implemented to avoid the aberrant proliferation of oncogene‐induced cells and orchestrate tissue repair in damaged areas. More than a decade ago, a collection of landmark articles clearly demonstrated that senescent cells are abundant in premalignant lesions in multiple tissues [128], laying the foundations for the concept of OIS *in vivo*. Such findings included the tumour‐prone phenotype of mice lacking crucial regulators of senescence (e.g., p53 and p16), being later expanded upon by other research groups using numerous genetically engineered mouse models of different cancer types. Senescence, like cancer, is a remarkably heterogeneous process depending on the trigger(s), cellular types, tissue context and other factors, and senescence heterogeneity appears to be particularly high in human tissue samples. This feature, alongside a research focus on precancerous lesions, has led to a very incomplete understanding of cellular senescence in patient samples when compared to preclinical or mouse models. Although senescence does appear to be abundant in patients' samples of preneoplastic tissue (e.g., preneoplastic prostate gland), more detailed studies in humans are required to dissect the extent, contexts and roles of cellular senescence. We must now tackle crucially important questions about senescence in human pathology, including understanding: (a) the role of secretion of a pro‐senescent SASP by senescent cells, whether and how this expands the arrest phenotype to nearby proliferative (oncogene‐activated) cells in specific neoplastic processes, and how this may go awry during tumour progression; (b) the contexts in which senescence contributes to tumour‐promoting effects, either by non‐cell‐autonomous (paracrine) and cell‐autonomous (senescence escape or reversion) activities; (c) which biomarkers can be used as signatures of these seemingly antagonistic processes. Critically, through assessment of the senescent burden, the identification of reliable biomarkers of senescence and of its associated effects at precancerous stages may provide valuable clinical information for prognosis and/or cancer early detection. As one example, precancerous lesions in human NSCLC include a spectrum of histopathological phenotypes that are thought to be progressive, atypical adenomatous hyperplasia (AAH), adenocarcinoma *in situ* (AIS) and minimally invasive adenocarcinoma (MIA) [244]. These are difficult to specifically define radiologically with conventional CT cross‐sectional imaging, and patients often have multifocal lesions in the lungs (often with non‐neoplastic lesions with similar imaging characteristics) where it is unclear which, if any, will lead to a frank cancer phenotype. Given the importance of OIS, it is now crucial that we fully characterise this process in these lesions, inform prognosis and apply our senescence detection tools (see Section 3) and novel senescence‐associated biomarkers to prognostically stratify patients and select lesions for local surgical/ablative therapy.

In addition to understanding the important interplay between senescence and (pre)neoplasia, systemic anticancer drugs and radiotherapy are in routine use in the management of both localised and advanced tumours. Both therapeutic types can induce, in addition to apoptosis, cancer cell senescence; however, as we have seen from Section 2, this is a double‐edged sword, potentially contributing both to tumour growth restriction and tumour progression. This particular area is poorly explored in human cancer, despite compelling evidence from preclinical models that suggests it will be an important contributor to treatment response and resistance. Availability of human tissue under appropriate contexts (essentially before and after senescence‐inducing treatment) is undoubtedly a limiting factor here, but we must now design protocols that allow this to be comprehensively and definitely assessed. In parallel, we must develop tools (for clinical use) to detect and monitor the senescent burden so that findings from such studies can be efficiently applied.

Of particular clinical interest are the senescent cancer cells that persist after cancer treatment (either micrometastatic cells or those comprising a subset of cells in a tumour mass), which present a risk for tumour recurrence and relapse [183] due to the pro‐inflammatory, pro‐tumorigenic, pro‐proliferative and immunosuppressive activities of the SASP [51, 150, 152]. Such detrimental consequences can also derive from therapy‐induced senescent stromal cells, which can bring about a variety of unwanted effects through altering the tumour (or even metastatic niche) microenvironment [174, 182, 183]. The intriguing possibility that therapy‐induced senescent cancer cells could re‐enter the cell cycle with an aggressive proliferative potential and cancer stem‐like properties has been recently reported [186], and may even explain clinical findings regarding progressive treatment resistance and tumour growth kinetics after successive lines of cytotoxic chemotherapy. These aspects require careful and urgent investigation in human cancer. Tools to track and monitor senescent cells would help provide baseline and longitudinal assessment of the response to different cancer treatment modalities, and evaluation of the associated risks of senescent phenotypes. Furthermore, they would help define populations of cells for targeting with senescence‐targeted drugs aimed at eliminating senescent cells and monitor their success. A number of studies using preclinical *in vivo* models have shown the efficiency of concomitant treatment combining senescence‐inducing therapeutics and senotherapies [198, 245, 246, 247].

A crucial hurdle in the development of novel diagnostic tools for senescence is the absence of a universal marker – this may reflect incomplete understanding (there is one/more and it/they have not yet been discovered) or intrinsic heterogeneity to the process (there is none, but certain biomarkers may define specific senescence phenotypes, perhaps being cell type‐, mutational context‐ or senescence trigger‐specific). The development of senolytics will be challenged by the same uncertainty, as well as their on‐target and off‐target effects which has hampered their translation [198]. A deeper understanding of the triggers, underlying molecular mechanisms and signalling pathways, as well as how they behave in different cell types and tissues will facilitate the identification and prioritisation of diagnostic and targetable biomarkers and the development of novel tools for detection and monitoring of senescent cells. Although a collection of senescence hallmarks has been compiled and some incredibly useful markers exist, we must now prioritise research into addressing knowledge gaps for the most immediate clinical utility. This includes the identification of specific, or differentially overexpressed, targetable surface markers at the level of the cell membrane (the so‐called senescent ‘surfaceome’), which could be used to design next‐generation senoprobes and/or more precise nanocarriers encapsulating tracers/contrasting agents/drugs for preferential delivery to senescent cells. So far, only a few surface proteins are observed to be overexpressed in senescent cells, including ICAM‐1 [248], NOTCH1 [59] NOTCH3 [249], DEP1 and B2MG [122, 250], and DPP4 [251]. These proteins are likely to be more enriched in particular senescent subtypes rather than being widespread. A recent study has identified the urokinase‐type plasminogen activator receptor (uPAR) as a more widely senescence‐induced cell surface protein [252]. Promisingly, in the same study, an immunotherapy approach based on uPAR‐specific CAR T cells efficiently ablated senescent cells *in vitro* and *in vivo*.

Longitudinal monitoring of senescent areas in clinical imaging remains a formidable challenge. To date, only a small number of senoprobes are capable of tracing senescent lesions in mouse models, with almost all being activated in cells exhibiting high SAβG activity, a broad, albeit imperfect, marker of senescent cells [217]. Most are based on modified and activatable fluorophores that are suitable only when low tissue penetrance is sufficient, markedly restricting their application to some murine models and to the human disease setting. The clinical use of senoprobes in deep‐tissue penetration bioimaging techniques will require specific adaptations, for instance by producing PET‐applicable senoprobes [229]. Significant limitations in cell specificity and penetration also apply to the current nanotechnologies targeting senescent cells – improved delivery strategies could encapsulate contrasting agents (e.g., gadolinium, for MRI detection) or radionuclides (e.g., F^18^, for PET detection). Senescence‐activatable nanocarriers could be used as theranostic devices, simultaneously detecting and eliminating premalignant lesions or the senescent cell burden after senescence‐inducing therapies. Biosafety concerns regarding the use of senoprobes and nanocarriers to monitor senescence should be addressed in parallel to ensure that this is not the bottleneck in translation. Preclinical studies in animal models must address optimal dosing; the mechanisms of cellular uptake and the intracellular trafficking; biocompatibility and biodistribution; the routes and kinetics of clearance. These will be necessary to move promising approaches into clinical studies.

Finally, the potential use of liquid biopsies (e.g., blood or even urine) to detect and assess the senescent burden would be a less invasive alternative to bioimaging techniques, although spatial resolution to pinpoint senescent populations would not be achievable using these alone. Senescent cancer and stromal cells are characterised by distinctive genomic (e.g., mutations) and/or epigenetic (e.g., DNA and histone methylation) modifications. Genomic modifications can be detected using cfDNA and, in combination with circulating SASP factors, used as biomarkers for early detection, prognosis and monitoring of cancer. This technology can be augmented by examining senescence methylation signatures, to give a fuller (systemic) view of the longitudinal tumour microenvironments, and early promising work in this field should now be built upon to refine this approach. Since senescent cells also secrete, as part of the SASP, a complex array of EVs and exosomes containing proteins, RNA, DNA and lipids that can be sampled using liquid biopsies, we must now determine their utility as a source of clinically relevant biomarkers. Small EVs secreted by senescent cells have been proposed to act as potent pro‐tumorigenic mediators [253, 254, 255] and may not therefore simply be by‐products of senescent cells, but provide more central (mechanistic) senescence biomarkers, perhaps with greater putative clinical relevance. In addition to these promising approaches, other techniques are emerging as promising modalities to assess senescence. Raman spectroscopy, a technique that allows determination of vibrational modes of molecules in cancer cells, has been used to study physical effects of biochemical deviations occurring in cancer [256]. Raman fingerprints have emerged as promising markers of cellular senescence, with ability to visualise changes occurring in the context of ageing of the skin and other tissues [257].

Given the important implications of senescent cell populations in cancer, as well as other age‐related pathologies, and the successful validation of multiple senotherapies in preclinical models *in vivo*, we anticipate a rapid expansion in this field and the development of novel and more specific tools and techniques to detect, monitor and target cellular senescence.

## 5 Concluding remarks

Cellular senescence is a defining feature of multiple types of precancerous lesions and responses to cancer therapies. Despite recent advances in the understanding of senescence biology in cancer and the development of tools to track senescent cells, numerous challenges must still be overcome to determine if cellular senescence can be exploited as a preventative, predictive, prognostic and/or diagnostic biomarker in cancer, and how this might be efficiently achieved. These include the need for (a) more specific, detectable and targetable molecular biomarkers of cellular senescence using preclinical model systems; (b) detailed studies of senescence using myriad human cancer tissues from different diagnostic and treatment contexts, determining the context‐specific biomarkers that should be prioritised for translational investigation; (c) a more detailed knowledge of, with deeper mechanistic insights into, how cellular senescence relates to different stages of precancerous lesions (e.g., metaplasia, dysplasia, hyperplasia, carcinoma *in situ*, minimally invasive carcinoma, etc.) and cancer (including mutational) contexts in diverse cancer types, and how senescence changes (biochemically, and at the levels of cell and tissue biology) in response to therapy (systemic agents and radiotherapy); (d) the development of more efficient tools to track senescent cells *ex vivo* in tissue and liquid biopsies, and *in situ* using bioimaging techniques; (e) the optimisation of diagnostic probes, sensors and therapies capable of targeting specifically senescent cells, including the preclinical validation of potential toxicities, biodistribution and timelines/routes for body elimination/excretion in animal models, and other important aspects currently limiting their translatability to human settings; and (f) the most promising diagnostic and therapeutic tools to enter well‐designed clinical trials to provide clinical proof of concept. The accelerating rate of progress in this field has been astounding, and we must now ensure that we focus on the strategies of highest clinical relevance and prioritise those with the greatest probability of successful translation to human cancer patients.

## Conflict of interest

The authors declare no conflict of interest.

## Author contributions

H‐LO and DM‐E conceptualized and wrote the manuscript. RH, GJD, and JEK reviewed the manuscript and provided expert opinion and critical insights. CG‐L contributed to the design of the figures and provided critical insights. All the authors commented on the text.
